# Long-term transcriptomic and proteomic effects in Sprague Dawley rat thyroid and plasma after internal low dose ^131^I exposure

**DOI:** 10.1371/journal.pone.0244098

**Published:** 2020-12-31

**Authors:** Malin Larsson, Nils Rudqvist, Johan Spetz, Emman Shubbar, Toshima Z. Parris, Britta Langen, Khalil Helou, Eva Forssell-Aronsson

**Affiliations:** 1 Departments of Radiation Physics, Institute of Clinical Sciences, Sahlgrenska Cancer Center, Sahlgrenska Academy at University of Gothenburg, Gothenburg, Sweden; 2 Departments of Oncology, Institute of Clinical Sciences, Sahlgrenska Cancer Center, Sahlgrenska Academy at University of Gothenburg, Gothenburg, Sweden; Helmholtz Zentrum München, GERMANY

## Abstract

**Background:**

Radioiodide (^131^I) is commonly used to treat thyroid cancer and hyperthyroidis.^131^I released during nuclear accidents, have resulted in increased incidence of thyroid cancer in children. Therefore, a better understanding of underlying cellular mechanisms behind ^131^I exposure is of great clinical and radiation protection interest. The aim of this work was to study the long-term dose-related effects of ^131^I exposure in thyroid tissue and plasma in young rats and identify potential biomarkers.

**Materials and methods:**

Male Sprague Dawley rats (5-week-old) were i.v. injected with 0.5, 5.0, 50 or 500 kBq ^131^I (D_thyroid_ ca 1–1000 mGy), and killed after nine months at which time the thyroid and blood samples were collected. Gene expression microarray analysis (thyroid samples) and LC-MS/MS analysis (thyroid and plasma samples) were performed to assess differential gene and protein expression profiles in treated and corresponding untreated control samples. Bioinformatics analyses were performed using the DAVID functional annotation tool and Ingenuity Pathway Analysis (IPA). The gene expression microarray data and LC-MS/MS data were validated using qRT-PCR and ELISA, respectively.

**Results:**

Nine ^131^I exposure-related candidate biomarkers (transcripts: *Afp* and *RT1-Bb*, and proteins: ARF3, DLD, IKBKB, NONO, RAB6A, RPN2, and SLC25A5) were identified in thyroid tissue. Two dose-related protein candidate biomarkers were identified in thyroid (APRT and LDHA) and two in plasma (DSG4 and TGM3). Candidate biomarkers for thyroid function included the ACADL and SORBS2 (all activities), TPO and TG proteins (low activities). ^131^I exposure was shown to have a profound effect on metabolism, immune system, apoptosis and cell death. Furthermore, several signalling pathways essential for normal cellular function (actin cytoskeleton signalling, HGF signalling, NRF2-mediated oxidative stress, integrin signalling, calcium signalling) were also significantly regulated.

**Conclusion:**

Exposure-related and dose-related effects on gene and protein expression generated few expression patterns useful as biomarkers for thyroid function and cancer.

## Introduction

The thyroid is an endocrine gland that produces iodine-containing hormones involved in metabolism and brain development. There are several isotopes of iodine, including radioactive ^131^I and ^123^I. Due to the natural accumulation of iodine in the thyroid, ^131^I (half-life 8.04 days [1]) has frequently been used to treat thyroid malignancies, including *e*.*g*. hyperthyroidism and thyroid cancer and it may be used in thyroid scintigraphic imaging. Also, ^131^I labelled radiopharmaceuticals, *e*.*g*. ^131^I-MIBG, used for treatment of other diseases contribute to irradiation of healthy thyroid tissue. In the clinic the absorbed dose to thyroid depends on the type of radiopharmaceutical, thyroid function, administered activity and age. According to the Swedish Radiation Safety Authority (2019), the absorbed dose to thyroid in adults was 0.063–7.2 Gy for diagnostics, and 60–2600 Gy for therapy [2, 3]. ^131^I-MIBG therapy resulted in around 2 Gy absorbed dose to thyroid in young children. Furthermore, ^131^I is one of the most commonly released radionuclides in nuclear weapon detonations and nuclear accidents. For medical use ^131^I is usually produced by (n, γ) reaction of ^130^Te to ^131^Te (half-life 25 min), which decays to ^131^I. Another way is by separation of fission products from ^235^U [4].

After the Chernobyl nuclear accident, individuals living in surrounding areas in Ukraine, Belarus, and Russia were initially exposed to ^131^I, and the absorbed dose to thyroid in children was 0–10 Gy, with the majority in the 0–5 Gy range and 1 Gy for pre-school children [5]. This resulted in significantly increased rates of primarily papillary thyroid cancer (PTC) in exposed children and adolescents [6–8]. Compared to adults, the biological uptake (concentration) of radioiodine is higher in young children due to the higher proliferation rate of the thyrocytes and the smaller thyroid size, which together with higher milk consumption by children may explain the absorbed dose to the thyroid in children after the Chernobyl accident. However, the increased absorbed dose to thyroid in children cannot explain the difference in thyroid cancer incidence when comparing children and adults [9]. Therefore, we need better knowledge of the effects of ionizing radiation on thyroid tissue, especially in young individuals. It is important to study both the short- and long-term radiobiological effects on thyroid tissue, since tumour initiation occurs early after exposure, while cancer diagnosis occurs years after exposure [10]. Exposure to ionizing radiation results in the activation/inhibition of several different cellular processes, where a number of the activated genes may have altered expression patterns [11, 12].

We have previously investigated changes in mRNA expression at early time points after ^131^I exposure in thyroid tissue samples from adult mice and rats using microarray techniques [13–16]. Several potential biomarkers were identified in thyroid tissue, specific for the administered activity level of ^131^I and the time after injection, e.g. *Klk1*, the *Klk1b* family, *Agpat9*, *Plau*, *Prf1*, and *S100a8*. Various biological functions were affected, and we showed in adult mice (6–8 mo.) effects related to cell adhesion 24 h after low-medium dose exposure to ^131^I [13]. In addition, calcium related gene changes and down regulation of parathyroid hormone and PPARG were found 24 h after ^131^I exposure of rat thyroid [14].

Our previous studies were performed on adult animals. It is thus necessary to perform radiobiological studies on young animals to evaluate age-related radiobiological effects. Taken together, transcriptome and proteome profiling may potentially be useful for biomarker discovery, as these techniques detect variations in gene expression over time for different stimuli, *e*.*g*. radiation.

The aim of the present work was to investigate biological effects associated with low to medium dose ^131^I exposure in young rats by studying changes in gene and protein expression in thyroid tissue and plasma, with special focus on exposure-related and dose-related effects and to identify potential biomarkers related to thyroid function alteration and cancer. Although a large number of regulated genes and proteins were identified, few demonstrated expression patterns (uniformly increased or decreased expression levels for all doses or expression levels increasing or decreasing with dose) useful as biomarkers for thyroid function and cancer.

## Material and methods

### Study design

Twenty young (5-week-old) male Sprague Dawley rats (Charles River Laboratories International, Inc., Salzfeld, Germany) were randomly divided into five groups containing four individuals each. Four groups were i.v. injected with 0.5, 5, 50 or 500 kBq ^131^I (GE Healthcare, Braunschweig, Germany) to obtain an absorbed dose to the thyroid of D_thyroid_ = 1, 10, 100 or 1000 mGy, respectively. One group was not treated and used as control. The animals were under daily supervision and received food (standard rat chow) and water *ad libitum*. The method for estimation of absorbed dose to the thyroid after i.v. injection of ^131^I has been described elsewhere [12]. In brief, the absorbed dose to thyroid was calculated using the Medical Internal Radiation Dose formalism [17]

$$D_{thyroid}={\tilde{A}}_{thyroid}*E\frac{\Phi }{m_{thyroid}}$$ (1)

$${\tilde{A}}_{thyroid}=A_{inj}\int_{0}^{t_{D}}a_{thyroid}(t)e^{-\lambda t}dt$$ (2)

where D_thyroid_ is the absorbed dose to the thyroid, Â is the cumulated ^131^I activity in thyroid, E is the mean emitted energy per disintegration, ***Φ*** is the absorbed fraction, m_thyroid_ is the thyroid mass, A_inj_ is the injected activity, a_thyroid_ is the activity in organs at time t, t_D_ is the time to which the absorbed dose contribution is calculated and **λ** is the radioactivity decay constant.

Nine months after ^131^I injection, the animals were killed by cardiac puncture under anaesthesia with pentobarbitalnatrium (APL; Kungens kurva, Sweden), followed by thyroid excision and collection of blood samples using heparin syringes. Thyroid samples were flash-frozen in liquid nitrogen and stored at -80oC until further processing. Plasma was collected from individual blood samples via centrifugation and stored at -80oC. The design of this study was approved by the Ethical Committee on Animal Experiments in Gothenburg, Sweden (Permit Number: 146–2015).

### Gene expression microarray analysis

Total RNA samples were extracted from flash-frozen thyroid tissue samples with the RNeasy Lipid Tissue Mini Kit (Qiagen; Hilden, Germany). Microarray analysis was performed at the Bioinformatics and Expression Analysis Core Facility at Karolinska Institute (Stockholm, Sweden) using Agilent Sureprint G3 Rat GE 8x60K microarrays (Agilent, Santa Clara, CA, USA). Nexus Expression 3.0 (BioDiscovery; El Segundo, CA, USA) was used to identify differentially expressed transcripts by comparing thyroid tissue from exposed and non-exposed rats. The fold change and FDR-adjusted p-value cut-offs (Benjamini-Hochberg method) were set to > 1.5, <-1.5 and < 0.01, respectively. Functional annotation data based on Gene Ontology (GO) terms with a p-value < 0.05 were compiled with Nexus Expression and associated with higher level cellular functions using an in-house model based on parental GO terms presented in Fig 5, including eight main categories and 31 subcategories (www.geneonology.org) [18]. Thyroid-specific genes were identified using the Human Protein Atlas database (www.proteinatlas.org) [19, 20]. The gene expression data discussed in this publication have been deposited in NCBI's Gene Expression Omnibus [21] and are accessible through GEO Series accession number GSE146051 (https://www.ncbi.nlm.nih.gov/geo/query/acc.cgi?acc=GSE146051).

### Liquid chromatography-tandem mass spectrometry (LC-MS/MS) analysis

#### Protein extraction and TMT (tandem mass tags) protein labelling

The thyroid tissue samples were homogenised in lysis buffer (25 mM Triethylammoinium bicarbonate, TEAB, 2% Sodium dodecyl sulfate (SDS)) using a FastPrep®-24 instrument (MP Biomedicals, OH, USA). Total protein concentration was determined with Pierce™ BCA Protein Assay (Thermo Fisher Scientific, Waltham, MA, USA). The samples were then group wise combined in equal amounts from each animal in the group. Tissue samples, 50 μg of total protein for each pooled group and 50 μg of a reference pool containing equal amounts of all samples, were reduced by DL-Dithiothreitol (DTT) and then trypsin (Pierce Trypsin Protease, MS Grade, Thermo Fisher Scientific, Waltham, MA, USA) digested using the filter-aided sample preparation (FASP) method modified according to Wisniewski *et al* [22].

Rat plasma samples were similarly combined per group and a total of 30 μl per pooled group was depleted of high-abundance proteins using the Seppro Rat spin column kit (Sigma). In short, samples diluted with 8 M urea were applied on Nanosep 30k Omega filters (Pall Life Sciences, Port Washington, NY, USA) and 8 M urea was used to repeatedly wash away the SDS. Alkylation was performed with methyl methane thiosulfonate (MMTS) diluted in digestion buffer (1% sodium deoxycholate (SDC), 20 mM TEAB) and the filters were repeatedly washed with digestion buffer. Trypsin in a ratio of 1:100 relative to protein amount was added with 25 mM TEAB and the samples were incubated in 37°C overnight. Another portion of trypsin was added the following morning and the samples were incubated for 4 h at 37°C. Peptides were subjected to isobaric mass tagging reagent TMT® according to the manufacturer’s instructions (Thermo Fisher Scientific, Waltham, MA, USA). In a set, each sample and a reference was labelled with a unique tag from a TMT 6plex or 10plex isobaric mass tag labelling kit. Following TMT labelling, the samples in a set were pooled and concentrated and acidified to about pH 2 to precipitate SDC.

The combined TMT samples were fractionated by basic-pH reversed-phase chromatography (bRP-LC) on an ÄKTApurifier (Amersham Pharmacia Biotech AB, Uppsala, Sweden) using a reversed-phase XBridge BEH C18 column (3.5 μm, 3.0x150 mm, Waters Corporation), 10 mM ammonium formate buffer at pH 10.0 as solvent A and 90% acetonitrile, 10% 10 mM ammonium formate at pH 10.0 as solvent B and the flow of 400 μl/min. Samples were separated into 27 fractions on a gradient elution from 0% to 7% B for 1 min, from 7% to 50% B for 32 min followed by an increase to 100% B for 1.5 min, and holding 100% B for 2 min. The fractions were evaporated and reconstituted in 15 μl of 3% acetonitrile, 0.2% formic acid for nLC-MS analysis.

#### Liquid chromatography-mass spectrometry analysis

The fractionated samples were analysed on an Orbitrap Fusion Tribrid mass spectrometer (Thermo Fisher Scientific) interfaced with an Easy-nLC 1000 liquid chromatography system (Thermo Fisher Scientific); solvent A was 0.2% formic acid (FA) in water and solvent B was 0.2% FA in acetonitrile. Peptides were trapped on an in-house packed 3 cm pre-column, and separated on an analytical column (75 μm x 30 cm, both backed with Reprosil-Pur C18 material, particle size 3 μm, Dr. Maisch) using the linear gradient from 5% to 25% B for 45 min, from 25% to 80% B for 5 min, 80% B for 10 min, at 300 nL/min flow rate.

Precursor MS scans were performed at 120 000 resolution, with the 4e5 AGC target in the *m/z* range 380–1200 with wide quadrupole isolation; the most abundant precursors with charges 2 to 7 were selected for fragmentation over the 3 s cycle time with the dynamic exclusion duration of 30 s. Precursors were isolated with 1.6 window, fragmented by collision induced dissociation (CID) at 30% collision energy with a maximum injection time of 70 ms and AGC target 1e4, and the MS2 spectra were detected in the ion trap followed by the synchronous isolation of the 5 most abundant MS2 fragment ions within *m/z* range of 400–900, and fragmentation by higher-energy collision dissociation (HCD) at the 55% collision energy; the resulting MS3 spectra were detected in the Orbitrap at 60,000 resolution in the *m/z* range 100–500 with the maximum injection time 120 ms and AGC target 1e5.

### Proteomic data analysis

Peptide and protein identification and quantification were performed using Proteome Discoverer version 1.4 (Thermo Fisher Scientific) with Mascot 2.3 or 2.5.1 (Matrix Science, London, United Kingdom) against the SwissProt database with taxonomy *Rattus norvegicus*, version 2015/04 (7935 sequences). Trypsin with no missed cleavage was used as a cleavage rule, precursor tolerance was set to 5 ppm and MS2 fragment tolerance was set to 500 mmu, monooxidation on methionine was set as a variable modification, cysteine methylthiolation, TMT-6 label on lysine and peptide N-termini were set as fixed modifications. Percolator was used for the peptide-spectrum match (PSM) validation with the strict false discovery rate (FDR) threshold of 1%.

The TMT reporter ions were identified in the MS3 HCD spectra with a mass tolerance of 3 mmu, and the resulting reporter abundance values for each sample were normalized on protein median in Proteome Discoverer 1.4. In total 3700 and 9747 peptides were found in plasma and thyroid tissue, respectively. Each protein was identified using 1–151 and 1–102 unique peptides for plasma and thyroid tissue, respectively.

The fold change cut-off were set to >1.5 and <-1.5, respectively. GO terms and the DAVID functional annotation tool (https://david.ncifcrf.gov/) were used for functional annotation of the LC-MS/MS data with a p-value cut off of <0.05, using modified Fisher´s exact test (EASE scores) and were categorised in the same manner as the transcripts, using the in-house model [23, 24]. Thyroid-specific proteins were identified using the Human Protein Atlas database (https://www.proteinatlas.org) [19, 20]. The mass spectrometry proteomics data have been deposited to the ProteomeXchange Consortium via the PRIDE [25] partner repository with the dataset identifier PXD017715.

### Ingenuity pathway analysis (IPA)

The Ingenuity Pathway Analysis software (IPA; Ingenuity Systems, Redwood City, USA) was used to analyse affected canonical pathways, biological functions and upstream regulators for both transcripts and proteins. The overlap between the experimental data and the Ingenuity Pathways Knowledge Base was determined using Fisher´s exact test (p<0.05). The z-score was used to determine the activation state of the upstream regulators; z>2 indicates activation, while z<-2 indicates inhibition.

### Validation of the microarray and the LC-MS/MS results

The *Slc25a5* gene and DSG4 protein were selected for validation of expression microarray and LC-MS/MS results, respectively. In order to validate the microarray results, qRT-PCR was performed on thyroid extracted mRNAs. In brief, the mRNAs were converted to cDNA using SuperScript™ VILO™ cDNA Synthesis Kit (Invitrogen, 11754–050), according to the manufacturer’s instructions. Primers corresponding to the *Slc25a5*, gene were run (Applied Biosystems) on a real-time 7500 HT sequence detection system (Applied Biosystems), with the SYBR green detection system (Applied Biosystems) The *beta-actin*, *Gapdh* and *HPTR1* genes were used as controls based on their uniform expression in thyroid tissue (Applied Biosystems). The relative expression of the analysed genes was determined in relation to the expression of the control genes using the ΔΔC_t_ method [14]. The intensity value was plotted against the ΔC_t_ value for each animal.

To validate the results from LC-MS/MS analysis, enzyme-linked immunosorbent assay (ELISA) was used for the DSG4 protein (MyBioSource Cat no; MBS9324066). In brief, standards and samples were prepared according to the manufacturer’s instructions, and were added into assigned wells. HRP conjugate was added to each well. The plate was incubated at 37 oC for one hour, and then washed according to the instructions. Chromogen solutions (A and B) was added to each well, followed by stop solution. Absorbance was measured for each well at 450 nm. The fold change value for each group was plotted against mean absorbed dose.

### Histological analysis

Thyroid tissue samples from each individual rat were fixed in formaldehyde and then imbedded in paraffin. Paraffin slices (4 μm) were stained using haematoxylin-eosin to evaluate thyroid tissue morphology, abnormal tissue structure, and the presence of tumour cells. The slides were evaluated by two certified pathologists.

## Results

### Regulated transcripts/proteins in thyroid tissue and plasma

**Fig 1:**
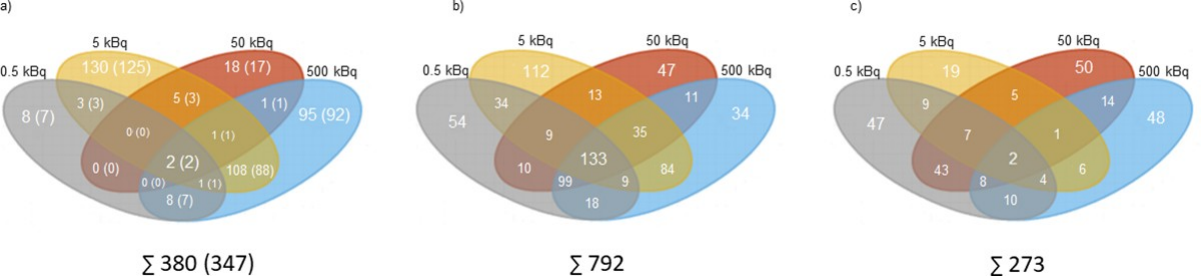
The number of statistically significant regulated transcripts and proteins in thyroid tissue and plasma 9 months after ^131^I administration in Sprague Dawley rats. Venn diagrams illustrating the distribution of the number of statistically significant a) regulated transcripts in thyroid tissue, with corresponding genes displayed in parentheses, b) expressed proteins in rat thyroid, and c) expressed proteins in rat plasma for 0.5 (in grey), 5 (in orange), 50 (in red) and 500 kBq (in blue).

**Fig 2:**
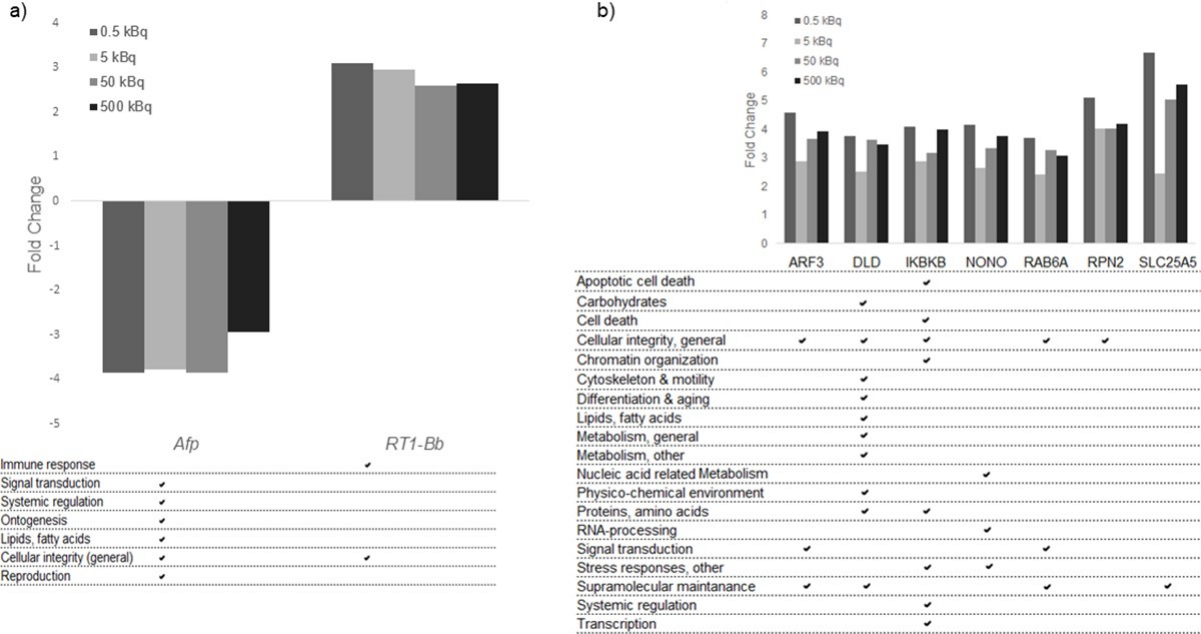
Exposure-related transcripts/proteins identified as candidate biomarkers in thyroid tissue, with their related GO terms. **a)** Transcript candidate biomarkers associated with response ^131^I exposure for all groups. Two transcripts were found in thyroid tissue. b**)** Seven protein candidate biomarkers for radiation exposure for all groups in thyroid tissue. Associated GO terms for each transcript and protein are displayed.

In thyroid tissue, the transcriptomic analysis revealed 380 differentially regulated transcripts (corresponding to 347 unique genes) associated with ^131^I exposure (Fig 1A). The majority of the identified transcripts (n = 251) were only found at one activity level (S1A Table), of which eight transcripts (seven genes), 130 transcripts (125 genes), 18 transcripts (17 genes), and 95 transcripts (92 genes) were identified for the 0.5, 5, 50 and 500 kBq group, respectively (S1 Table). Only two transcripts (*Afp and Rt1-Bb)* were regulated at all activity levels (Fig 2A).

The proteomic analysis identified 792 and 273 differentially expressed proteins associated with ^131^I exposure in thyroid tissue (Fig 1B) and plasma (Fig 1C), respectively. In the thyroid, 247 proteins were regulated at only one activity level, including 54, 112, 47 and 34 proteins identified in the 0.5, 5, 50 and 500 kBq groups, respectively (S1B Table). Furthermore, 133 proteins were regulated at all activity levels (Table 1). Of the 273 proteins differentially regulated in plasma, 164 were only found at one activity level (S1C Table) (47, 19, 50 and 48 proteins were regulated in the 0.5, 5, 50 and 500 kBq groups, respectively) and two proteins (TMSB4X and SPP1) were regulated at all activity levels (Table 1).

**Table 1:** Proteins in thyroid tissue with differential expression levels above or below 1.5 for all activity groups (0.5, 5, 50 and 500 kBq) (totally 133 proteins). The 20 most abundant proteins for each activity group are marked in grey. Eight of these proteins were present in all four groups (ARF3, DLD, IKBKB, KTR4, NONO, RAB6A, RPN2 and SLC25A5). For the majority of proteins, the expression levels were ether increased or decreased for all activity levels.

Proteins found in thyroid tissue with significant expression levels for all four groups	0.5 kBq	5 kBq	50 kBq	500 kBq
AARS	1.97	1.55	1.79	1.90
ABHD14B	3.54	2.67	2.24	2.77
ACADL	1.99	2.49	1.96	1.56
ACADM	1.89	2.12	1.81	1.67
ACADS	2.43	1.98	2.43	2.14
ACADVL	1.55	2.00	1.79	1.86
ACLY	4.00	2.88	3.26	2.26
ACO2	2.14	1.68	2.71	2.10
ALDH2	2.98	2.36	2.97	4.00
ALDH3A2	2.84	1.73	3.19	2.30
ALDH5A1	2.53	2.79	2.42	2.49
ALDH6A1	1.72	1.75	1.73	2.24
ALDH9A1	2.59	2.34	2.52	2.25
APMAP	3.13	1.52	2.42	2.79
APOE	-1.94	-1.65	-1.73	-1.60
APRT	1.75	1.99	1.59	1.98
ARCN1	2.01	1.55	1.76	2.01
ARF3	4.57	2.86	3.66	3.91
ARPC2	2.45	1.54	2.25	2.04
ATP2A1	3.45	2.31	3.00	2.71
ATP5A1	1.98	2.32	2.70	2.49
BST2	-1.76	-1.67	-1.53	-1.55
CAP1	2.7	1.82	1.79	2.40
CASQ1	-2.19	-1.61	-1.62	-1.70
CASQ2	-4.77	-1.56	-1.86	-2.33
CCT3	1.83	1.76	1.90	1.86
CD99L2	-1.75	-1.70	-1.73	-1.52
CDC42	2.52	2.09	2.12	2.14
CDH2	-1.68	-1.83	-1.62	-1.91
CHMP4C	-2.32	-1.54	-1.65	-1.97
CMA1	1.79	1.73	1.62	1.81
COQ6	1.70	2.10	1.67	1.81
CPQ	3.69	1.87	1.92	2.83
CRYL1	2.59	2.22	1.61	2.24
CS	1.73	2.16	1.73	1.82
CSNK2A1	2.24	2.21	2.54	2.20
DDR1	2.61	2.35	1.93	2.57
DECR1	1.65	1.62	1.53	1.91
DHRS4	-3.77	-2.37	-2.26	-2.74
DLAT	2.48	2.53	2.39	2.43
DLD	3.77	2.53	3.64	3.47
DNAJC5	-2.67	-1.79	-1.88	-2.15
DPP3	2.31	2.29	1.80	2.06
DPP7	2.55	1.58	1.78	2.50
DPYSL2	3.11	1.72	1.94	2.56
ECI1	1.94	2.53	1.73	1.77
EEF1A2	2.91	1.78	3.24	2.66
EEF1G	1.93	1.50	1.80	2.03
EEF2	2.05	1.52	1.60	1.83
EEF2K	-1.66	-1.91	-2.66	-2.33
EIF2S1	2.07	1.60	2.11	1.93
EZR	2.60	1.68	2.26	2.36
FABP4	2.33	2.00	1.96	2.07
FASN	3.02	4.31	2.37	1.56
GALM	3.47	1.88	2.91	2.94
GAPDH	2.11	1.78	2.38	2.05
GDI2	3.46	2.58	2.40	2.69
GLUL	2.18	1.64	1.65	1.64
GNAI2	2.11	2.09	1.50	1.87
GOT2	1.56	2.35	2.24	2.46
GPI	1.56	1.73	1.76	1.69
GYPC	-2.15	-1.84	-1.75	-2.70
HADH	2.11	2.30	1.85	1.58
HADHA	2.84	2.17	3.03	2.48
HADHB	2.13	1.77	2.61	1.79
HNRNPA1	3.59	1.82	3.50	3.65
HNRNPM	2.18	2.07	1.66	2.22
IKBKB	4.10	2.89	3.16	3.98
IVD	2.92	2.15	2.46	3.19
KLHL41	1.66	1.64	1.80	1.69
KRT4	-4.27	2.91	4.15	-4.12
LDHA	2.23	2.04	2.43	1.80
MARCKS	-1.75	-1.70	-1.71	-1.56
MCPT1	5.16	1.72	4.56	4.42
MLEC	5.24	2.06	4.68	5.50
MYBPC1	3.07	2.24	3.92	2.93
NAMPT	1.57	1.68	1.51	1.87
NAP1L4	-1.75	-1.62	-1.51	-1.65
NDUFAF4	-2.42	-1.70	-2.14	-2.59
NOL3	-2.64	-2.01	-1.75	-1.78
NONO	4.14	2.63	3.33	3.75
NUDT5	1.74	1.59	1.62	1.61
NUP54	2.09	1.95	1.65	2.17
OXCT1	1.64	2.16	1.57	1.96
PDHB	2.56	3.05	2.45	2.27
PEPD	1.89	1.53	1.55	2.17
PFN1	2.04	1.68	1.74	1.89
PHB2	2.68	2.40	2.86	2.57
PIR	1.96	1.80	1.90	1.89
PKM	2.78	2.31	3.09	1.98
POR	2.41	1.77	2.03	2.10
PRDX3	1.80	2.30	1.55	1.75
PRPSAP2	2.58	2.21	2.26	2.25
PSMA2	1.60	1.82	1.52	1.80
PSMA4	2.84	2.50	2.55	2.74
PSMA7	1.79	1.57	1.57	1.78
PSMB3	2.45	2.07	2.32	2.65
PSMB4	2.55	2.11	2.31	2.51
PTGR1	2.98	2.15	2.17	3.07
PYGM	2.90	1.61	3.93	2.11
QDPR	1.90	1.83	1.75	1.74
RAB11B	2.30	2.17	1.98	2.03
RAB18	1.92	1.60	1.71	1.61
RAB2A	3.43	2.14	3.56	3.04
RAB6A	3.69	2.42	3.26	3.06
RAC1	2.41	2.15	2.04	2.15
RAN	2.70	1.71	2.83	2.27
RAP1A	2.27	1.81	1.83	2.22
RPL11	2.20	1.55	1.93	2.16
RPL3	4.75	1.70	3.92	3.55
RPL9	3.94	2.02	4.02	3.17
RPN2	5.12	4.03	4.01	4.17
RT1-BB	2.22	1.52	2.17	1.75
SAE1	-2.10	-1.86	-2.11	-2.37
SCN1B	-2.58	-1.91	-1.51	-2.06
SEC11A	3.87	2.14	3.41	3.23
SELENBP1	2.81	1.86	2.03	2.61
SEPT2	2.82	2.03	2.37	2.87
SEPT7	2.29	1.52	2.04	2.18
SLC25A5	6.68	2.46	5.05	5.55
SORBS2	1.92	1.64	2.06	1.76
SPTBN2	2.30	2.22	1.56	2.15
SRPRB	2.22	2.20	1.61	2.02
TKT	3.57	2.73	2.90	2.43
TMED10	3.08	1.98	2.18	2.98
TMED2	2.73	1.77	2.29	2.59
TNNI2	-2.08	-1.56	-1.51	-1.78
TOR1AIP1	-1.85	-1.98	-1.84	-1.50
TPM2	-2.81	-1.53	-1.68	-1.99
TUFM	1.93	1.72	1.99	2.05
UQCRC2	1.88	2.46	2.17	2.05
VDAC1	1.70	1.57	2.18	1.94
YBX3	-2.13	-2.02	-1.50	-1.93
**Proteins in plasma with significant expression levels for all four groups**	**0.5 kBq**	**5 kBq**	**50 kBq**	**500 kBq**
TMSB4X	-2.30	1.62	-1.73	1.51
SPP1	1.65	1.89	-2.02	1.57

### Commonly detected proteins in thyroid tissue and plasma

Interestingly, 63 proteins were commonly regulated in both thyroid and plasma for the same group (Table 2). The majority of the differentially regulated proteins were found in the 500 kBq and 0.5 kBq groups and were, in general, either over- or underexpressed in both thyroid and plasma samples, irrespective of administered activity. A number of proteins were identified with similar expression patterns in both thyroid and plasma, including 20 proteins in the 0.5 kBq group, (overexpression: ACO2, ALDH2, APRT, 12CA1, CA2, EEF1a1, GPNMB, H1f0, HIST1h4b, LGALS5, PEPD, PSMA4, PSMB3, and TG; underexpression: BASP1, BID, BIN2, CLIP2, RT1-AW2 and TPM4), five proteins in the 5 kBq group (overexpression: DPP7 and PRDX2; underexpression: BASP1, CRYAB and S100A6), 13 proteins in the 50 kBq group (overexpression: ACLY, ALDH2, FASN, GDA, GDI2, GPDI, PEPD, PSMB3, PSMB4 and RAN; underexpression: HRSPI2, PVALB and SMPX), and 15 proteins in the 500 kBq group (overexpression: ALDH2, COMP, ESYT1, HSP90ab1, PDIA4, PSMA4, PSMB3 and TSMB4X; underexpression: APOC1, BASP1, CRSP3, DBI, LGALS1, MB and MCAM).

**Table 2:** Expression of statistically significant regulated proteins in both thyroid and plasma. The figure displays all 63 proteins that were statistically significantly regulated in both thyroid tissue and plasma for the same activity level (0.5, 5, 50 or 500 kBq) nine months after ^131^I injection. The fold change cut-off was set to ±1.5. A positive or negative fold change is represented by + and–, respectively. If there was a difference between direction of regulation in thyroid and plasma, the thyroid data is presented first and then the plasma data, separated by /. Associated GO terms are displayed for each protein.

					GO terms							
Proteins	0.5 kBq	5 kBq	50 kBq	500 kBq	Cell cycle and differentiation	Cell communication	Cellular integrity	DNA integrity	Gene expression integrity	Metabolism	Organismic regulation	Stress responses
RT1-AW2	**-**											
BID	**-**											
CLIP2	**-**											
TPM4	**-**											
PFN1	**+/-**											
BIN2	**-**											
GSTP1	**+/-**											
RAB7a	**+/-**											
CAP1	**+/-**											
RAC1	**+/-**											
MCPT1	**+/-**	**+/-**	* *	*+/-*								
BASP1	**-**	**-**	* *	*-*								
SEPT7	**+/-**		* *	* *								
ALDH2	**+**		*+*	*+*								
PEPD	**+**		*+*	* *								
HIST1h4b	**+**		* *	* *								
EEF1a1	**+**		* *	* *								
PSMA4	**+**		* *	*+*								
PSMB3	**+**		*+*	*+*								
H1f0	**+**		* *	* *								
CA2	**+**		* *	* *								
ACO2	**+**		* *	* *								
CA1	**+**		* *	* *								
APRT	**+**		* *	* *								
TG	**+**		* *	* *								
GPNMB	**+**		* *	* *								
MYL3	**-/+**		* *	* *								
LGALS5	**+**		* *	* *								
S100A6		**-**	* *	* *								
CRYAB		**-**	* *	* *								
HSPE1		**-/+**	* *	* *								
PRDX2		**+**	* *	* *								
DPP7		**+**	* *	* *								
PPIF		**-/+**	* *	* *								
CPQ			+/-	+/-								
SMPX			-									
PVALB			-									
ATP5a1			+/-									
HRSP12			-									
MDH2			+/-									
GPD1			+									
PSMB4			+									
FASN			+									
RAN			+									
GDI2			+									
GDA			+									
ACLY			+									
FABP4				+/-								
CSRP3				-								
LGALS1				-								
SELENBP1				+/-								
DBI				-								
MB				-								
GOT1				+/-								
MCAM				-								
APOC1				-								
TMSB4X				+								
COMP				+								
TPM2				-/+								
HSP90ab1				+								
PDIA4				+								
MYL1				-/+								
ESYT1				+								

### Exposure-related candidate biomarkers

Potential biomarkers for ^131^I exposure, irrespective of injected ^131^I activity, were selected from the transcriptomic and proteomic data as having a uniform increase or decrease in expression levels in all four groups. In thyroid tissue, the *Afp* and *RT1-Bb* transcripts were commonly regulated in all four groups, where *Afp* expression was consistently down-regulated and *RT1-Bb* up-regulated, but with no clear activity-related response (Fig 2A).

The majority of the 133 differentially regulated proteins identified in thyroid tissue had a uniform increase or decrease in protein expression level in the four groups, and are thus candidates for exposure related biomarkers useful to determinate if an individual is exposed or not, irrespective of dose (Table 1). Seven proteins found in thyroid tissue (ARF3, DLD, IKBKB, NONO, RAB6A, RPN2, and SLC25A5) had elevated expression level with fold change > 2.0 (Fig 2B). No protein with homogenous expression levels was obtained in all four groups for plasma.

For the three groups with the lowest activities (0.5–50 kBq), six proteins found in thyroid tissue (AGMN, APOD, CAPZA2, GSTZ1, FAM213A, RBM20) and five proteins in plasma (CYP27B1, DSG4, RRAS, TAOK3, TGM3) were identified as potential exposure biomarkers (S2 Table). The corresponding candidate biomarkers for the intermediate activity groups (5–500 kBq) in thyroid included one transcript (*Ankrd2*) and 31 proteins found in thyroid tissue (AKR1A1, APOA2, ARMC10, ATP6V1B2, CCT2, CDNF, DSTN, GATC, HBA1, Haemoglobin subunit beta-2, HSDL2, IL1RAP, IRGC, KNG1, KRT13, KRT15, LXN, MAPK3, MUSTN1, NRADD, PARVA, PYURF, RPL18, SEPT8, SLC3A2, SVIP, TNNI1, UPF0729 protein C18orf32 homolog, USMG5, VAT1, VCP) (S2 Table). The corresponding candidate biomarkers for the groups with the highest activities (50–500 kBq) included one thyroid transcript (*Cd300lg*), eleven protein found in thyroid tissue (BLOC1S2, CALCOCO1, COMP, Ester hydrolase C11orf54 homolog, GMFB, HBB, IDH2, LDHB, NDUFS2, PSMC2, YBX1) and eleven proteins in plasma (ATRN, CHGA, CPQ, CTBS, CYCS, ESYT1, F7, LTA4H, PF4, PVALB, SMPX) (S3 Table).

### Dose-related candidate biomarkers

**Fig 3:**
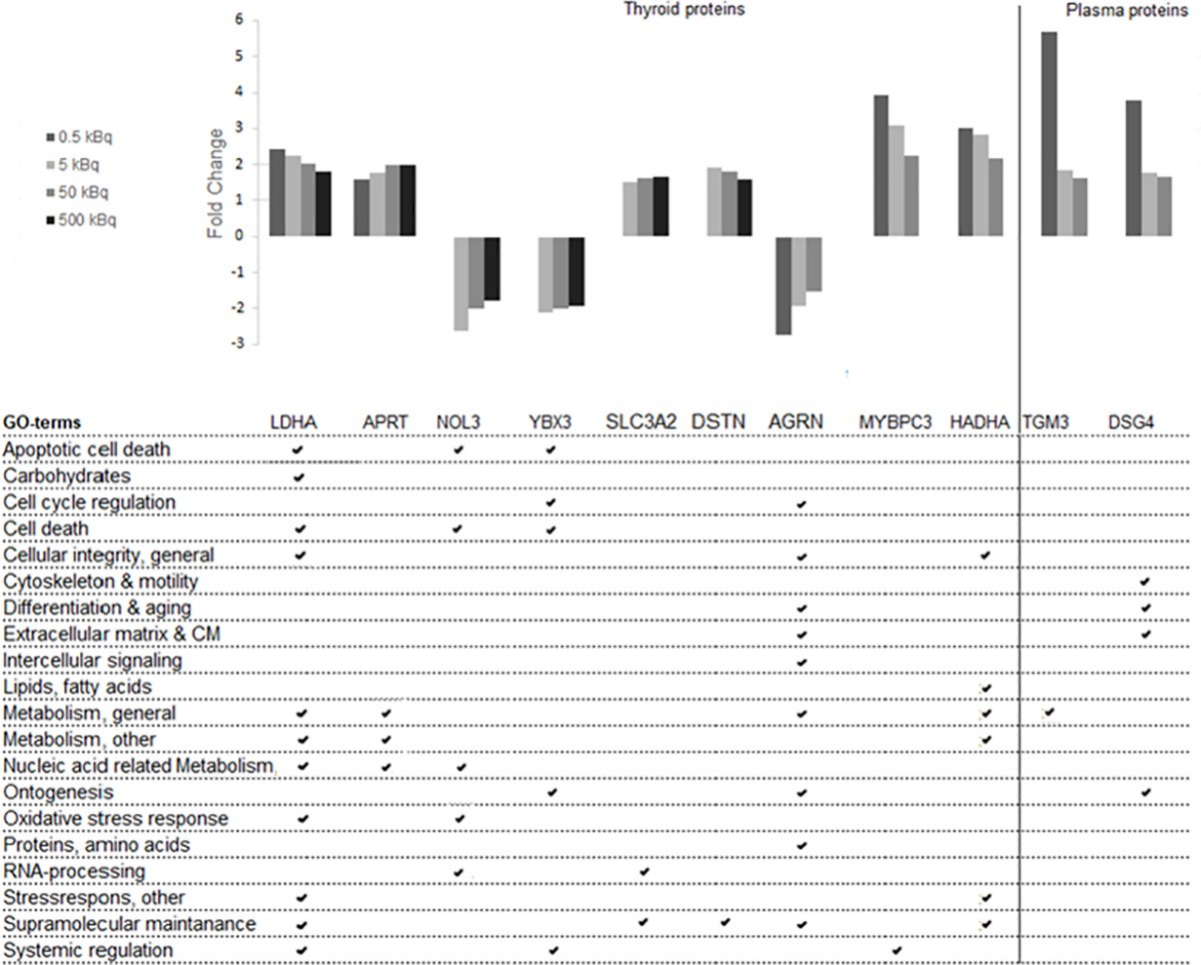
Regulated dose-related candidate biomarker proteins from thyroid and plasma, and their related GO terms. Protein candidate biomarkers with dose-related response for at least three of the test groups are presented. Nine proteins were found in thyroid tissue and two proteins in plasma.

To identify dose-related biomarkers, we analysed common transcripts or proteins with a monotonic dose-response relationship (either increasing or decreasing expression levels for all groups) in all four or in three of the groups (0.5–50 kBq and 5–500 kBq). No differentially regulated transcripts with dose-response features were found in the transcriptome analysis of rat thyroid tissue. In contrast, the LDHA and APRT proteins were significantly overexpressed for all four groups in a dose-dependent manner in the proteomic analysis of thyroid tissue (Fig 3). Furthermore, seven dose-related proteins were identified in three of the groups (0.5–50 kBq or 5–500 kBq). For the three highest activities (5–500 kBq), two proteins with underexpression (NOL3 and YBX3) and two proteins with overexpression, (SCL3A2 and DSTN) were identified. For the three lowest activities (0.5–50 kBq) AGRN was underexpressed, while two proteins were overexpressed (MYBPC3 and HADHA). In plasma, the TGM3 and DSG4 proteins were overexpressed in a dose-dependent manner for the three lowest activities (0.5–50 kBq).

### Genes/proteins associated with thyroid function and thyroid cancer in thyroid tissue and plasma

The Human Protein Atlas database was used to identify regulated genes and proteins associated with thyroid function or thyroid cancer (Table 3). In thyroid tissue, five genes were found (*Bhlhe41* (5 kBq), *Calca* (0.5 kBq), *Calcb* (0.5 and 500 kBq), *Dio2* (5 kBq), *Krt7* (500kBq). In thyroid tissue, seven proteins (ACADL (all groups), CLIP2 (0.5 and 500 kBq), SORBS2 (all groups), BCL2 (0.5 kBq group), CALCA (500 kBq group), TG (0.5–5 kBq), and TPO (5 and 500 kBq)) were detected. In plasma, four proteins were found (CLIP2 (0.5 and 50 kBq), CYP27B1 (0.5–50 kBq), PLCB4 (0.5 and 50 kBq), and TG (0.5 and 500 kBq)).

**Table 3:** Genes/proteins associated with thyroid function and thyroid cancer for all groups in thyroid tissue and plasma in young rats injected with 0.5–500 kBq ^131^I. The fold change for each transcript/protein is presented in parenthesis.

Thyroid function and thyroid cancer associated genes/proteins				
	0.5 kBq	5 kBq	50 kBq	500 kBq
Thyroid transcripts				
	*Calca* (-1.81)	*Bhlhe41* (-1.65)		*Krt7* (-4,76)
	*Calcb* (-1.87)	*Dio2* (-2.22)		*Calcb* (-2.24)
Proteins found in thyroid tissue				
	ACADL (1.96)	ACADL (1.99)	ACADL (2.49)	ACADL (1.56)
	CLIP2 (-1.58)			CLIP2 (-1.51)
	SORBS2 (2.06)	SORBS2 (1.92)	SORBS2 (1.64)	SORBS2 (1.76)
	TG (2.03)	TG (2.66)		
		TPO (2.38)		TPO (1.71)
	BCL2 (-1.64)			CALCA (-2.18)
Plasma proteins				
	CLIP2 (-1.91)		CLIP2 (-1.72)	
	CYP27B1 (1.68)	CYP27B1 (1.55)	CYP27B1 (1.57)	
	PLCB4 (2.08)		PLCB4 (1.96)	
	TG (2.32)			TG (3.17)

### Validation of methods and histological evaluation of thyroid samples

**Fig 4:**
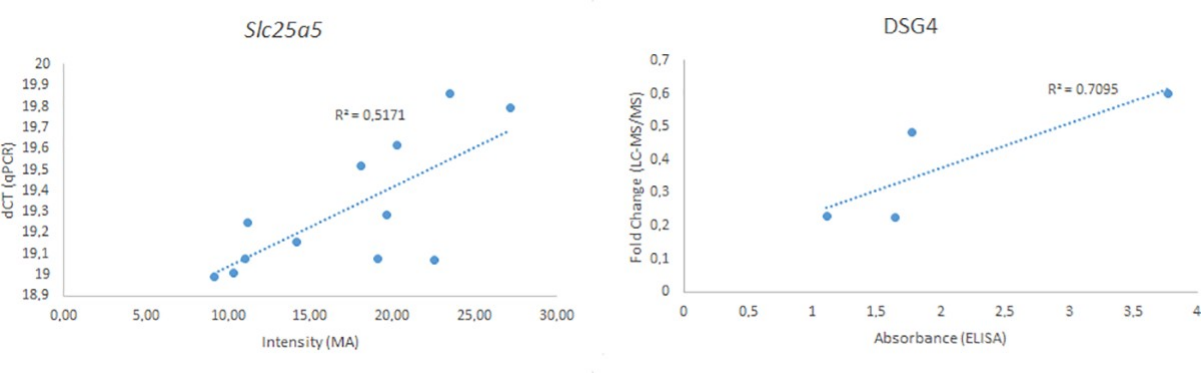
Validation of microarray and LC-MS/MS methods using the Slc25a5 gene (left) and DSG4 protein (right). The intensity from the microarray of the Slc25a5 gene for each animal is displayed against the ΔCT value from the RT-qPCR analysis. The fold change value for the DSG4 protein was plotted against the absorbance from the ELISA analysis. The R2 value was calculated using linear regression.

The validation of microarray data using RT-qPCR on extracted thyroid mRNA for the *Slc25a5* gene revealed a correlation (R2 = 0.52). (Fig 4). The validation of the LC-MS/MS analysis using ELISA of the DSG4 protein demonstrated a significant correlation (R2 = 0.71) (Fig 4).

Three of the sixteen thyroid tissue samples from exposed animals showed in some parts abnormal tissue structures (neoplastic tissue).

### Gene ontology annotations

**Fig 5:**
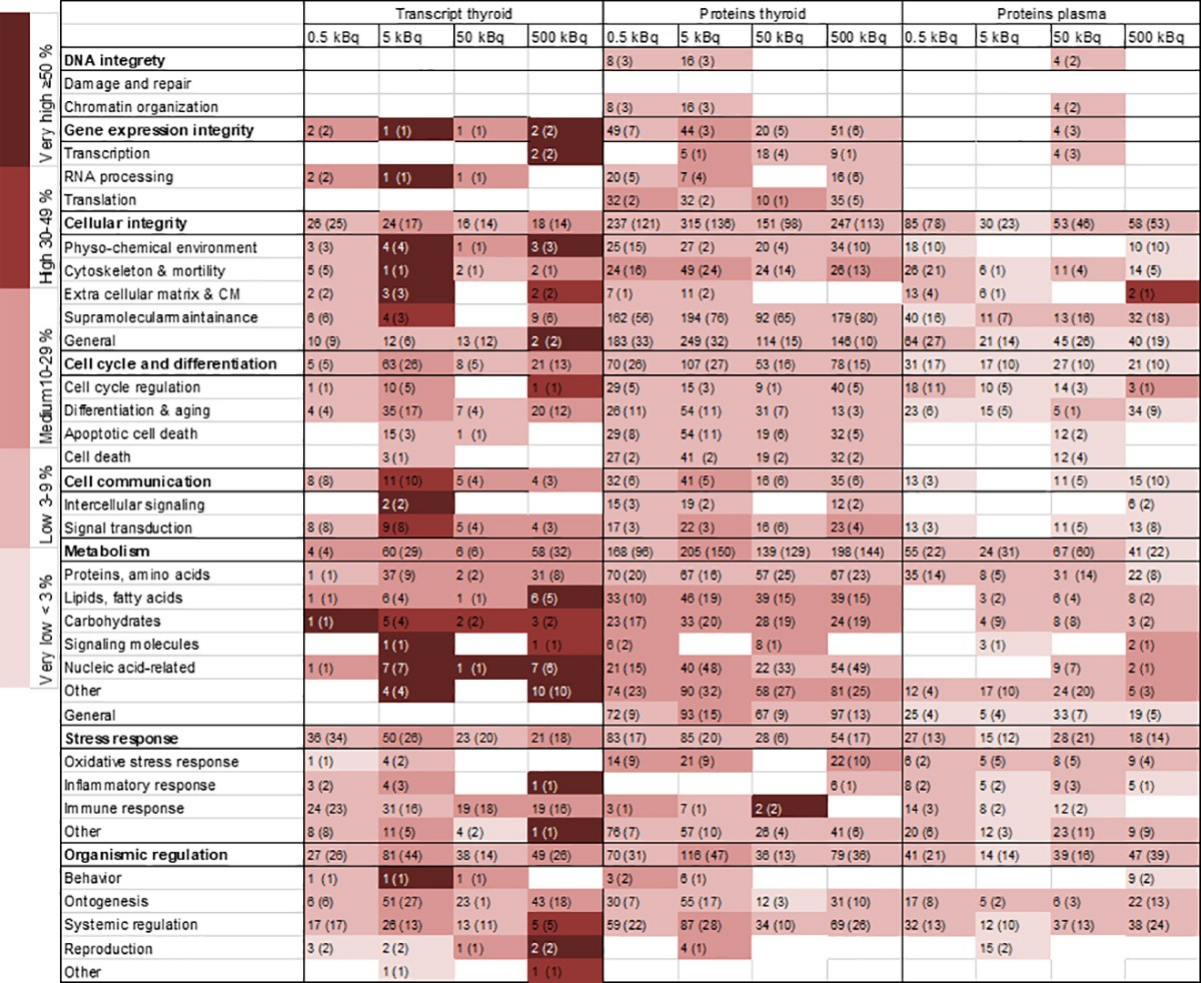
Heat map of enriched biological processes categorised after cellular function for regulated transcripts and proteins in thyroid tissue and plasma. Biological processes were enriched from significantly regulated transcripts/proteins (based on GO terms) and then grouped into categories and subcategories of associated cellular function based on GO ancestor charts (http://geneontology.org). The number of scored transcripts or proteins are displayed, together with the total number of associated GO terms presented in parentheses. The percentage of scored vs. filtered transcripts/proteins, related to each cellular function, is displayed by colour according to the colour bar.

Enriched biological processes for thyroid transcripts (BioDiscovery Nexus Expression 3.0 analysis) and proteins detected in thyroid and plasma tissues (DAVID functional annotation tool) were categorised into GO terms associated with cellular function and the extent of regulation calculated for each GO term (Fig 5). A clear association with specific GO terms was obtained for the thyroid transcripts and the highest number of associated terms was seen for proteins found in thyroid tissue. Some of the groups differed from the rest, *e*.*g*. proteins found in thyroid tissue were predominantly associated with apoptosis, suggesting cellular damage. However, the main cellular functions were similarly affected for all tissues, with metabolism (nucleic acid-related response) displaying the most pronounced response to ^131^I irradiation. Less pronounced response to irradiation was shown for DNA integrity.

Gene Ontology association analysis was also performed for transcripts and proteins identified as candidate biomarkers. The analysis showed that the *Afp* and *RT1-Bb* exposure-related transcripts were associated with signal transduction, systemic regulation, ontogenesis, lipid metabolism, cellular integrity and reproduction, and cellular integrity and immune response, respectively (Fig 2A). The GO terms associated with the seven exposure-related protein candidates were mainly associated with cellular integrity (general) and supramolecular maintenance (Fig 2B). The associated GO terms for the eleven proteins in thyroid and plasma related to dose were mainly involved in apoptotic cell death, cell death, cellular integrity, metabolism, nucleic acid related metabolism, ontogenesis, supramolecular maintenance and systemic regulation (Fig 3). Furthermore, the majority of the proteins obtained in both thyroid tissue and plasma for the same activity level were involved in cellular integrity and metabolism (Table 2).

IPA was used to analyse canonical pathways, functions and diseases and upstream regulators for transcripts and proteins in thyroid and proteins in plasma. In general, significantly affected canonical signalling pathways, functions and diseases and predicted upstream regulators were predominantly associated with proteins found in thyroid tissue, with pathway activation (z-score ≥2) being most prevalent (Tables 4–6). Pathway activation/inhibition was common for proteins in plasma in the group with the lowest activity (0.5 kBq). The majority of the signalling pathways were only significantly affected in one of the groups (Table 4). Several other signalling pathways were common in two groups, including actin cytoskeleton signalling, B cell receptor signalling and HGF signalling (0.5 and 50 kBq groups), integrin signalling (5 and 50 kBq groups) and calcium signalling (50 and 500 kBq groups). The NRF2-mediated oxidative stress response was common in three groups (5, 50 and 500 kBq). For the biological functions and diseases analyses, the majority of activated functions were seen in the 5 and 50 kBq groups and the total number of associated functions was similar for two groups (Table 5). Few up-stream regulators were significantly regulated, with only one or two for each group. Only the PPARG was identified as an up-stream regulator in all four groups (Table 6).

**Table 4:** Predicted canonical signalling pathways affected in rat thyroid and plasma after exposure to low doses of ^131^I. All analyses were performed for all twelve samples and the only obtained canonical signalling pathway for transcripts was “Dopamine Receptor Signalling” for the 0.5 kBq group. The direction of regulation of each target molecule is marked by **bold** for increased expression and underscore for decreased expression.

Ingenuity Canonical Pathway	p	z	Target molecules in dataset		
**0.5 kBq** **Proteomics (Thyroid Tissue)**					
Actin Cytoskeleton Signaling	7.9·10^–4^	2.2	MYL2,MYLK2,**MYH7**,**VCL**,MYL3,ACTN1		
**Proteomics (Plasma)**					
B Cell Receptor Signaling	1.5·10^–3^	-2.4	RAP1B, RAP2B, CFL1,RRAS, RAC1, LYN		
Fc Epsilon RI Signaling	8.9·10^–3^	-2.0	RRAS, RAC1,LYN, PRKCA		
HGF Signaling	8.9·10^–3^	-2.0	RAP1B, RRAS, RAC1,PRKCA		
**5 kBq**					
**Proteomics (Thyroid Tissue)**					
NRF2-mediated Oxidative Stress Response	3.2·10^–4^	2.6	**AKR7A2**,**GSTA3**,**PPIB**,**PRDX1**,HSPB8,**DNAJC3**,**KRAS**,**AKR1A1**,DNAJC5, **DNAJB11**,**MAPK3**,**CAT**,**VCP**,ACTC1,**GSTP1**		
Integrin Signaling	1.1·10^–3^	2.1	**ACTR2**,**PARVA**,**PFN1**,MYL2,**RALB**,**RAC1**,**KRAS**,**GSN**,**RAP1A**,**ARF3**, **ARPC2**,**MAPK3**,ARF4,**ITGB4**,ACTC1		
β-Adrenergic Signaling	3.5·10^–2^	2.2	**GNAI2**,**PYGM**,**MAPK3**,**RACK1**,**PYGB**,**KRAS**		
**50 kBq** **Proteomics (Thyroid Tissue)**					
Integrin Signaling	7.4·10^–5^	2.1	**IKBKB**,**CSNK2A1**,**MAPK1**,**MAPK3**,**RAC1**,**KRAS**,**DDR1**		
Fcγ Receptor-mediated Phagocytosis in Macrophages and Monocytes	6.5·10^–5^	2.8	**PARVA**,**PFN1**,**MAPK1**,**ARF3**,**ARPC2**,**MAPK3**,**ACTB**,WASL,**RAC1**,**CRK**, **KRAS**,**RAP1A**		
Renal Cell Carcinoma Signaling	1.5·10^–4^	2.4	**GNAI2**,**MAPK1**,**EZR**,**ACTB**,WASL,**RAC1**,**CRK**,**RAP1A**		
Calcium Signaling	8.5·10^–4^	2.2	TNNI2,**MAPK1**,**MAPK3**,CASQ1,TNNI1,**RAP1A**,TPM2,CASQ2,**ATP2A1**		
Signaling by Rho Family GTPases	8.7·10^–4^	2.5	**MAPK1**,**MAPK3**,**RAC1**,**KRAS**,**DDR1**		
Ephrin Receptor Signaling	1.1·10^–3^	2.3	**AKR1A1**,**SOD2**,DNAJC5,**MAPK1**,**MAPK3**,**ACTB**,**VCP**,**KRAS**		
PTEN Signaling	1.6·10^–3^	-2.6	**GNAI2**,**PYGM**,**MAPK1**,**MAPK3**,**KRAS**		
Actin Cytoskeleton Signaling	1.6·10^–3^	3.0	**IKBKB**,**MAPK1**,**MAPK3**,**RAC1**,**KRAS**		
RhoA Signaling	1.7·10^–3^	2.6	**CSNK2A1**,**MAPK1**,**MAPK3**,**CRK**,**KRAS**		
NGF Signaling	1.7·10^–3^	2.6	**MAPK1**,**MAPK3**,**RAC1**,**KRAS**,**RAP1A**		
STAT3 Pathway	4.2·10^–3^	2.2	**GNAI2**,**MAPK1**,**MAPK3**,CASQ1,**RAC1**		
Paxillin Signaling	6.2·10^–3^	2.4	**MAPK1**,**ARPC2**,**MAPK3**,**RAC1**,**KRAS**		
NRF2-mediated Oxidative Stress Response	6.8·10^–3^	2.2	**GNAI2**,**MAPK1**,**MAPK3**,**RAC1**,**KRAS**		
β-Adrenergic Signaling	8.5·10^–3^	2.2	**IKBKB**, CDH2,**MAPK1**,**MAPK3**,**KRAS**		
fMLP Signaling in Neutrophils	8.5·10^–3^	2.4	**GNAI2**,**MAPK1**,**ARPC2**, **MAPK3**,WASL,**RAC1**,**CRK**,**KRAS**,**RAP1A**		
LPS-stimulated MAPK Signaling	8.9·10^–3^	2.2	**MAPK1**,**MAPK3**,**RAC1**,**CRK**,**KRAS**,**FH**,**RAP1A**		
Leukocyte Extravasation Signaling	1.0·10^–2^	2.1	**PARVA**, **MAPK1**,**ACTB**, **RAC1**, **CRK**, **KRAS**		
PDGF Signaling	1.1·10^–2^	2.2	**GNAI2**,**MAPK1**,**ARPC2**,**MAPK3**,**RAC1**,**KRAS**		
PKθ Signaling in T Lymphocytes	1.1·10^–2^	2.4	**IKBKB**,**MAPK1**,**MAPK3**,**RAC1**,**KRAS**		
p70S6K Signaling	1.3·10^–2^	2.4	**GNAI2**,**MAPK1**,**EEF2**,**MAPK3**,**KRAS**, EEF2K		
HGF Signaling	2.8·10^–2^	2.2	**GNAI2**,**MAPK1**,**MAPK3**,**RAC1**, **CRK**, **KRAS**		
Sphingosine-1-phosphate Signaling	2.9·10^–2^	2.2	**GNAI2**,**IKBKB**, **MAPK1**,**MAPK3**,**KRAS**		
Rac Signaling	2.9·10^–2^	2.2	I**KBKB**,**MAPK1**,**MAPK3**,**RAC1**,**KRAS**,**RAP1A**		
CXCR4 Signaling	3.4·10^–2^	2.4	**GNAI2**,**SEPT8**,CDH2,**MAPK1**,**ARPC2**,**EZR**,**MAPK3**,**ACTB**,**SEPT7**,**RAC1**, **SEPT2**		
GNRH Signaling	4.1·10^–2^	2.2	**SEPT8**,**PFN1**,**ARPC2**,**EZR**,**ACTB**,**SEPT7**,**SEPT2**		
Gα12/13 Signaling	4.1·10^–2^	2.2	**IKBKB**,**MAPK1**,**MAPK3**,**RAC1**,**CRK**,**KRAS**,**RAP1A**		
Role of NFAT in Regulation of the Immune Response	4.3·10^–2^	2.4	**MAPK1**,**RAB11B**,**ARPC2**,**EZR**,**MAPK3**,**ACTB**,**RAC1**,**CRK**		
B Cell Receptor Signaling	4.7·10^–2^	2.4	KNG1,**PFN1**,**MAPK1**,**ARPC2**,**EZR**,**MAPK3**,**ACTB**,**RAC1**,**CRK**,**KRAS**		
**500kBq** **Proteomics (Thyroid Tissue)**					
Calcium Signaling	1.9·10^–6^	2.1	TNNI2,MYL2,CHP1,**PRKAR2A**, TPM1,TPM2,**RAP1A**,MYL1,**ATP2A1**, PPP3R1,**MAPK3**,CASQ1,TNNI1,CASQ2,MYL3,**CAMK2G**		
NRF2-mediated Oxidative Stress Response	3.1·10^–2^	2.2	**AKR7A2**,**AKR1A1**,DNAJC5,**PPIB**,**MAPK3**,**CAT**,**VCP**,**DNAJC3**,**GSTP1**		

**Table 5:** Diseases or functions annotation of thyroid and plasma using IPA. Four, six, six and one diseases or functions were seen for 0.5, 5, 50 and 500 kBq groups, respectively. All diseases or functions were activated except for “fatty acid metabolism” and “apoptosis”. All but three diseases or functions, “fatty acid metabolism”, “neuronal cell death” and “apoptosis” were obtained in the thyroid proteomic analysis. The direction of regulation of each target molecule is marked by **bold** for increased expression and underscore for decreased expression.

Diseases or Functions Annotation	P	z	Target molecules in dataset
**0.5 kBq** **Proteomics (Thyroid Tissue)**			
differentiation of connective tissue cells	1.3·10^–3^	2.2	BCL2,**CA2**,**CEBPA**,NUCB2,**POR**,**RACK1**
**Proteomics (Plasma)**			
fatty acid metabolism	1.0·10^–3^	-2.2	**CEACAM1**,FABP1,GNAI3,LYN,**PAM**,RAC1,RGN
neuronal cell death	2.7·10^–2^	2.2	**A2M**,ILK,**NPM1**,**SRC**,**TGM1**,YWHAB
**5 kBq** **Proteomics (Thyroid Tissue)**			
metabolism of protein	9.7·10^–10^	2.4	**ACY1**,**AMBP**,Apoc1,APOE,**CMA1**,**CPQ**,**CTSC**, **CTSD**,**DDX39B**,**DPP3**,**DPP7**,**EEF2**,**EIF2S1**, EIF4EBP1,**GAPDH**,**GSN**,**H2AFZ**,**HSPA5**,HSPB8, Kng1/Kng1l1,**MAPK3**,PAM,**RACK1**,**RPL10A**, **Rps3a1**,**TPP1**,UCHL1
synthesis of protein	6.1·10^–5^	2.0	**DDX39B,EEF2**,**EIF2S1**,EIF4EBP1,**GSN**,**H2AFZ**, **HSPA5**,HSPB8,**MAPK3**,**RACK1**,**RPL10A**,**Rps3a1**
proliferation of cells	7.1·10^–4^	2.2	ADA,ADK,AKAP12,APOE,**CAT**,**Cdc42**,CDH2, **CMA1**,**COL1A1**,CRYAB,**CST3**,**CTNNB1**,DCN, **DPYSL2**,EIF4EBP1,**EZR**,**FABP4**,GAP43,**GLUL**, **GNAI2**,GRN, **IKBKB**, KNG1,Kng1/Kng1l1,**KRAS**, **Ldha/RGD1562690**,LGALS1,LUM,**MAPK3**, **NAMPT**,NDRG2,NDUFAF4,NOL3,**PLG**,**POR**,**PPT1**,**PRDX3**,**RAC1**,**Rps3a1**,S100A4,SERPINH1, **STMN1**,**TF**,Tpm2,TPT1
cell death of muscle cells	8.7·10^–4^	2.4	CRYAB,**CTNNB1**,**EEF1A1**,**GAPDH**,HSPB1, HSPB6,HSPB8,HSPE1,**KRAS**, **NAMPT**, NOL3, **RAC1**
cell death of cardiomyocytes	1.2·10^–3^	2.0	CRYAB,**GAPDH**,HSPB1,HSPB6,HSPB8,HSPE1, **KRAS**,**NAMPT**,NOL3
**Proteomics (Plasma)**			
apoptosis	3.6·10^–5^	-2.2	CRYAB,**SPP1**,**PPIF**,RRAS,ANXA1,**HSPE1**,RTN4,**OSMR**,CTH,**PRDX2**
**50 kBq** **Proteomics (Thyroid Tissue)**			

metabolism of protein	1.2·10^–4^	2.2	APOE,**CMA1**,**CPQ**,**DPP3**,**DPP7**,**EEF2**,**EIF2S1**, **GAPDH**,**MAPK1**,**MAPK3**,**PTH**,RIDA
DNA replication	3.8·10^–3^	2.0	**Cdc42**,Hmgb1,**MAPK1**,**RAC1**
differentiation of cells	4.8·10^–3^	2.4	AGRN,**CAPZB**,**Cdc42**,CDH2,**CRK**,**DDR1**,**DPYSL2**,**GDA**,**GLUL**,Hmgb1,**KRAS**,**MAPK1**,NOL3,**OPA1**, **POR**,**RAC1**
synthesis of protein	4.4·10^–3^	2.2	**EEF2**,**EIF2S1**,**MAPK1**,**MAPK3**,**PTH**,RIDA
differentiation of pheochromocytoma cell lines	1.4·10^–2^	2.2	**CRK**,**DDR1**,**KRAS**,**MAPK1**,**RAC1**
metabolism of DNA	1.6·10^–2^	2.2	**Cdc42**,Hmgb1,**MAPK1**,**PFN1**,**RAC1**
**500 kBq** **Proteomics (Thyroid Tissue)**			
metabolism of protein	6.0·10^–11^	2.1	**ACY1**,**AMBP**,**ANPEP**,Apoc1,APOE,**CAPN2**,CHP1,**CMA1**,**CPQ**,**CTSC**,**CTSD**,**DDX39B**,**DPP3**,**DPP7**, **EEF2**,**EIF2S1**,**GAPDH**,**GSN**,**MAPK3**,PCSK2,**PTH**,**RACK1**,**RPL10A**,**Rps3a1**,**TPP1**,UCHL1

**Table 6:** Upstream regulators for thyroid and plasma using IPA. PPARG was activated in all four groups (0.5–500 kBq) found in thyroid tissue protein analysis. TSC2 was activated for the 0.5 kBq group found in protein analysis of plasma. In the protein analysis of thyroid tissue TNF and NFKB (complex) were inhibited and activated for the 5 and 50 kBq group, respectively. The direction of regulation of each target molecule is marked by **bold** for increased expression and underscore for decreased expression.

Upstream regulators	p	z	Target molecules in dataset
**0.5 kBq** **Proteomics (Thyroid Tissue)**			
PPARG	1.2·10^–7^	3.0	**GSTM5**,**GSTP1**
**Proteomics (Plasma)**			
TSC2	1.1·10^–3^	2.0	**A2M**,**ANXA2**,**CRYAB**,**GPNMB**
**5 kBq** **Proteomics (Thyroid Tissue)**			
PPARG	2.6·10^–6^	3.0	CHGB
TNF	1.6·10^–2^	-2.0	AGRN,APOE,CASQ2,CD59,**COMP**,F3,FABP5,**SOD2**,YBX3
**50 kBq** **Proteomics (Thyroid Tissue)**			
PPARG	4.8·10^–10^	3.1	**FABP5**,**KRT19**,**Ldha/RGD1562690**,**SOD2**
NFkB (complex)	3.6·10^–2^	2.0	COL1A1
**500 kBq** **Proteomics (Thyroid Tissue)**			
PPARG	4.8·10^–7^	3.0	**PTPN11**

## Discussion

In this study, we investigated long-term (9 months) effects of low to medium dose ^131^I exposure on expression patterns in thyroid tissue and plasma collected from young rats. The gene and protein expressions were compared with corresponding data from non-exposed controls of the same batch and age. Then the expression patterns were compared with absorbed dose, and potential relation to thyroid function and cancer were examined for the identified genes and proteins. However, biomarker discovery is not easy as ideal biomarkers must fulfil several criteria. Biomarkers for exposure should give unidirectional differential expression levels, while biomarkers for dose relationships should have monotonic differential expression levels that correlate to absorbed dose. For thyroid function, a biomarker needs to be thyroid-specific and dependent on cellular response. Lastly, tumour biomarkers must be a) detectable at an early stage in cancer development, b) absent in healthy individuals, and c) tumour marker abundance should correlate directly with tumour size [26]. In addition, biomarkers should also ideally be detectable in body fluids, which would simplify sample collection and be less invasive for patients.

In total, we found nine potential exposure-related biomarkers in thyroid (transcripts: *Afp*, *RT1-Bb*, and proteins: ARF3, DLD, IKBKB, NONO, RAB6A, RPN2 and SLC25A5), and eleven differentially expressed dose-related proteins (in thyroid; AGRN, APRT, DSTN, HADHA, LDHA, MYBPC3, NOL3, SLC3A2 and YBX3; in plasma; DSG4 and TGM3). The *Afp* gene had lower expression after ^131^I exposure in thyroid. *Afp* is highly expressed in foetal liver cells, foetal yolk sac and the gastrointestinal tract [27]. The *Afp gene* is also involved in the regulation of cellular growth and its mRNA has been detected in both human PTC and in normal thyroid [28]. Elevated expression of AFP has been found in radiation-induced hepatocellular carcinomas [29]. The *RT1-Bb* gene had higher expression after ^131^I exposure in thyroid, and belongs to the MHC II class of proteins that are involved in the immune system [30]. All seven exposure-related proteins in thyroid tissue were overexpressed after irradiation. The ARF3 protein is GTP binding and involved with protein trafficking and vesicle binding, the *Arf3* has also been suggested as a marker in radiation transformed breast cancer [31, 32]. The DLD protein is a part of several multi-enzyme complexes involved in energy metabolism, and it has also been proposed as a biomarker for UV radiation induced skin aging [32, 33]. IKBKB has an important role in the NF-kappa beta signalling pathway, which is triggered by radiation and can cause resistance for irradiated cancer cells [34]. NONO is an RNA binding protein that is part of transcription and RNA splicing, and has been associated with UV radiation induced cellular damages [35, 36]. RAB6A is located in the Golgi apparatus and is involved in the targeting and fusion of transport carriers [32, 37]. RPN2 is a membrane protein that mediates protein translocation in the endoplasmic reticulum and the SLC25A5 protein transports ADP from the cytoplasm to the mitochondria and ATP in the opposite direction [32].

All eleven dose-related proteins, nine in thyroid tissue and two in plasma, were overexpressed after ^131^I exposure. Among those, the most promising response relationship was found for LDHA and APRT in thyroid tissue (0.5–500 kBq) and TGM3 and DSG4 in plasma (0.5–50 kBq). LDHA catalyses the last step in aerobic glycolysis and is primarily located in muscle tissue, and APRT is an enzyme involved in the adenine nucleotide metabolism [32]. Furthermore, LDHA overexpression has been found in BRAF V600E-mutated thyroid cancer and inactivation of this protein can make cells more sensitive to radiation [38, 39]. TGM3 is involved in the transformation of the cell envelope in epidermis and hair follicles, but it is also a tumour suppressor and has been proposed as a progression marker for radio-chemotherapy of head and neck cancers [32, 40]. DSG4 is involved in cell adhesion, primarily in epithelial cells [32].

As far as we know, data on short- or long-term effects on rats after ^131^I exposure have not been published. When comparing our present long-term data in young rats with publicly available short-term data in adult mice, few common differentially expressed transcripts and proteins were identified [13–16]. Interestingly, the *Klk1* (0.5 kBq thyroid), CPA3 (0.5 kBq thyroid), and S100A8 (50 kBq plasma) markers were commonly identified in both the present study and previous studies on the short-term effects of ^131^I exposure on thyroid. *Klk1*, CPA3 and S100A8 are important for thyroid function in both mice and rats [19, 20]. Genome homology between humans and rodents is high (almost 85% at the expression level) and they share several cellular mechanisms [41]. We believe that comparing results generated between species despite differences in species, age and time is valuable for the transfer of the generated results to humans. Furthermore, identified markers between species with similar function are conserved during evolution reflect important function. Therefore, the commonly identified markers in rats and previously in mice adult and young as well as persist with time after exposure initiation, might be potential exposure-related biomarkers also for humans.

The ACADL and SORBS2 protein, were regulated at all dose levels studied and are the most promising candidate biomarkers, since they have been previously associated with thyroid function [19, 20] Among the thyroid-specific proteins, elevated levels of thyroid peroxidase (TPO) were found in the three groups with lowest administered activities (0.5–50 kBq). This finding is in contrast to results from a study using ^60^Co gamma irradiated cultured human thyroid epithelial cells, where TPO expression levels were reduced, possibly indicating short-term effects *in vitro* [42]. Since the TPO and TG thyroid associated proteins were differentially expressed in some of our exposed groups (primarily at low doses), these proteins might be potential biomarkers for exposure at low dose-levels since no monotonic dose-response relationships were found.

In total, five transcripts (*Bhlhe41*, *Calca*, *Calcb*, *Dio2* and *Krt7*) related to thyroid function were found in some of the groups. *Bhlhe41* is related to circadian rhythm [32]. The *Calca* gene codes for calcitonin production, which is involved in the calcium regulation and phosphor metabolism [32]. *Calcb* is a paralog to *Calca* and is associated with medullary thyroid carcinoma [32]. The *Dio2* (Deiodinase 2) gene is highly associated with thyroid function and encodes for a protein that catalyses the deiodination of T4 (thyroxine) to the active thyroid hormone T3 (triiodothyronine). The DIO2 protein is also found in several tissue types and is thought to be a local producer of T3 [32]. Overexpression of the *Dio2* gene has been seen in follicular adenoma and Grave´s disease [32]. The *Krt7* gene belongs to the keratin family and stimulates DNA synthesis in cells by blocking interferon-dependent interphase [32].

In total, eight proteins associated with thyroid function were identified in thyroid tissue and/or plasma (ACADL, BCL2, CALCA, CYP27B1, PLCB4, SORBS2, TG and TPO). The ACADL and SORBS2 proteins were overexpressed in all groups in thyroid tissue. ACADL is an enzyme involved in the metabolism of fatty acids and amino acids, while SORBS2 belongs to a family of non-receptor protein-tyrosine kinases [32]. Thyroglobulin (TG) and thyroid peroxidase (TPO) are thyroid-specific proteins. TG is a protein primarily produced by the thyroid gland, involved in the production of the iodine-containing hormones T3 and T4, and the storage of T4 [32]. TG is clinically relevant, especially to predict thyroid residues, since TG blood levels are related to thyroid function, rather than thyroid malignancy [43]. Elevated TG expression levels can therefore indicate that thyroid function is impaired after exposure. TPO is an enzyme essential for thyroid gland function involved in T4 and T3 production, and mutations in this gene can lead to hyperthyroidism [32]. Of the observed proteins, the strongest association with thyroid function was seen for TG and TPO. These proteins were not differentially regulated in all groups, but upregulated at the two lowest activities (0.5 and 5 kBq) and at the highest activity (500 kBq). This is interesting since it suggests that thyroid function after irradiation is not only affected by dose, but other biological effects as well, as previously suggested iodine deficiency, age at exposure, gender and the issue with increased screening [44].

For clinical use, biomarkers in plasma would be preferable, and only four proteins were differentially expressed regulated among those known to be related to thyroid function (TG, CYP27B1, PLCB4 were overexpressed and CLIP2 was underexpressed). TG is interesting, but is a protein that might be influenced by general physiological status. CYP27B1 is involved in transforming vitamin D to its active form, and PLCB4 is an enzyme, which transduces signals over the plasma membrane by using second message molecules diacylglycerol (DAG) and inositol 1,4,5-trisphosphate (IP3), both of which have functions that are important for normal thyroid and body function [32]. *CLIP2* has been proposed as a biomarker for thyroid cancer in several studies on children in the Chernobyl cohort [45–47]. In the present study, we identified decreased CLIP2 expression in exposed thyroid tissue and plasma (all doses), while Selmansberger *et al*. showed an increase in both protein and mRNA expression levels in thyroid tumour tissue in children [48].

The higher association of transcripts than proteins with GO terms is most probably explained by investigational bias, since transcriptomic profiling is hitherto far more common than proteomic analyses, with less data for protein enrichment. A pronounced association with metabolism, immune system, apoptosis and cell death was established for all samples. Increased metabolism is caused by increased thyroid hormone production, and might suggest abnormal thyroid function (hyperthyroidism). The strong association with apoptosis and cell death may be due to thyroid cell impairment and function. The immune system is highly involved in cancer and other diseases. All these effects indicate that thyroid cells are affected by irradiation (observed at as low as 1 mGy), and that the thyroid function differs from that of non-irradiated thyroid.

The significant canonical signalling pathways that were commonly identified in more than one group are all necessary for functions related to cellular survival. NRF2-oxidative stress leads to the binding of antioxidant responsive elements and can thus neutralise reactive molecules [32]. The upstream regulator PPARG is involved in differentiation, cell growth and nuclear regulation. It is primarily found in adipose tissue, is associated with diabetes 2 and colon cancer but is also related to follicular thyroid cancer and abnormal thyroid function [32, 49]. It is also interesting to note that activation of PPARG was not influenced by dose-level. We also previously showed downregulation of PPARG expression in our short-term studies on rats [14].

Other studies on ^131^I-induced effects *in vitro* and *in vivo* have focused on specific effects on non-coding RNAs and selected proteins. Long non-coding RNA MEG_3_ has been a suggested as a biomarker for thyroid cancer and ^131^I radio-resistance [50]. Proteomic studies on PTC tissue from humans have suggested NTRK1, metalloproteinases (MMP-1, MMP-9 and MMP-13) and Cathepsins (-W and -X), NF-KB and other apoptosis associated proteins as radiation-induced PTC biomarkers, together with SPANXA as an important protein for thyroid cancer development [51–56]. However, none of these were found in the present study. *In vitro* studies on thyroid cells have shown that DNA damage after ^131^I exposure increases in the presence of high levels of TSH [57]. Thyroid function has also been evaluated, by measurements of the IL-6 TNF-αand NO proteins in serum samples using a rat model using four week old rats irradiated with 5.5 MBq ^131^I [58]. Some of these proteins were found also in the present study, but the expression levels were not in the same direction (increased or decreased), probably due to the longer time after administration and the much lower amount of ^131^I administered.

In the present study, several regulated genes and proteins for exposure and dose-response are presented. However, no single biomarker was found that seems to fulfil the necessary criteria for being an optimal biomarker related to dose response, with clearly differentiated expression level. These findings are in line with our previous studies on the short-term effects of exposure to ionizing radiation in adult mice and rats, where no clear dose-response relationships were found for any single transcript [14, 59]. We and others then suggested the use of a panel of several genes to evaluate radiation response [11, 14, 16]. This situation seems to also be the case for long-term effects in young rats.

## Conclusions

In the present study, the gene and protein expression profiles for rat thyroid tissue and plasma were investigated in young rats nine months after exposure to low amounts of ^131^I. The results showed that only a few genes or proteins altered their expression in a clear dose-related manner. Expression patterns mostly demonstrated altered (up and down) regulation at certain dose levels in a non-monotonic manner. The most promising dose-related candidate biomarkers were the LDHA and APRT proteins (in thyroid), and TGM3 and DSG4 proteins (in plasma, only for low doses). The most promising exposure-related candidate biomarkers were the *Afp* and *RT1-Bb* transcripts in thyroid, both showing uniform differential expression patterns, irrespective of dose level.

The GO term association analysis revealed that most of the regulated genes/proteins were associated with immune response, which is influenced in many diseases, including cancer. For the transcripts, a pronounced association with cellular processes related to cell cycle regulation, metabolism and cancer was found.

Thyroid function-related proteins overexpressed in thyroid and/or plasma included SORBS2, ACADL, TG, and TPO (low doses). CLIP2, which has previously been found to be overexpressed in children with PTC in the Chernobyl cohort, was underexpressed in thyroid and plasma in the present study on young rats.

Preferably, biomarker examinations should be performed in blood samples for less invasive tissue sampling. However, in the present study the majority of the candidate biomarkers were found only in thyroid tissue, and thyroid needle biopsies are routinely collected clinically and thus possible to collect.

In conclusion, this work resulted in increased knowledge of late radiation-induced cellular effects after low to medium dose exposure from ^131^I. Novel candidate biomarkers related to exposure or absorbed dose were identified and previously proposed candidate biomarkers were not detected in this work. The identified biomarkers need further validation using human tissue samples.

## Supporting information

S1 Table Uniquely regulated a) transcripts in thyroid tissue b) proteins in thyroid tissue and c) proteins in plasma for each group. (DOCX)

S2 Table Transcripts and proteins in thyroid and plasma tissue with statistically significant differential expression levels for three of four groups. (DOCX)

S3 Table Transcripts and proteins in thyroid and plasma tissue with statistically significant differential expression levels for two out of four groups. (DOCX)
