# Correction: Long-term transcriptomic and proteomic effects in Sprague Dawley rat thyroid and plasma after internal low dose 131I exposure

**DOI:** 10.1371/journal.pone.0259429

**Published:** 2021-10-27

**Authors:** Malin Larsson, Nils Rudqvist, Johan Spetz, Emman Shubbar, Toshima Z. Parris, Britta Langen, Khalil Helou, Eva Forssell-Aronsson

There is an error in Table 2. The vales in the “GO terms” columns were accidentally left blank. The corrected Table 2 is shown below.

**Table 2:** Expression of statistically significant regulated proteins in both thyroid and plasma. The figure displays all 63 proteins that were statistically significantly regulated in both thyroid tissue and plasma for the same activity level (0.5, 5, 50 or 500 kBq) nine months after 131I injection. The fold change cut-off was set to ±1.5. A positive or negative fold change is represented by + and–, respectively. If there was a difference between direction of regulation in thyroid and plasma, the thyroid data is presented first and then the plasma data, separated by /. Associated GO terms are displayed for each protein.

Proteins	0.5 kBq	5 kBq	50 kBq	500 kBq	Cell cycle and differentiation	Cell communication	Cellular integrity	DNA integrity	Gene expression integrity	Metabolism	Organismic regulation
RT1-AW2	**-**					X					
BID	**-**				X		X				
CLIP2	**-**						X				
TPM4	**-**										X
PFN1	**+/-**						X				X
BIN2	**-**						X				
GSTP1	**+/-**						X			X	
RAB7a	**+/-**					X	X				X
CAP1	**+/-**						X			X	
RAC1	**+/-**				X	X	X			X	X
MCPT1	**+/-**	**+/-**	* *	*+/-*						X	
BASP1	**-**	**-**	* *	*-*							
SEPT7	**+/-**		* *	* *	X		X			X	
ALDH2	**+**		*+*	*+*			X		X	X	X
PEPD	**+**		*+*	* *						X	
HIST1h4b	**+**		* *	* *			X	X			X
EEF1a1	**+**		* *	* *	X				X		
PSMA4	**+**		* *	*+*	X		X			X	
PSMB3	**+**		*+*	*+*	X		X			X	
H1f0	**+**		* *	* *			X	X			
CA2	**+**		* *	* *			X				X
ACO2	**+**		* *	* *						X	
CA1	**+**		* *	* *			X				
APRT	**+**		* *	* *						X	X
TG	**+**		* *	* *			X				
GPNMB	**+**		* *	* *			X				X
MYL3	**-/+**		* *	* *							
LGALS5	**+**		* *	* *						X	
S100A6		**-**	* *	* *	X						
CRYAB		**-**	* *	* *	X		X			X	X
HSPE1		**-/+**	* *	* *			X				
PRDX2		**+**	* *	* *	X		X	X			
DPP7		**+**	* *	* *						X	
PPIF		**-/+**	* *	* *	X		X				
CPQ			+/-	+/-						X	X
SMPX			-								X
PVALB			-				X				
ATP5a1			+/-		X		X			X	
HRSP12			-		X		X			X	
MDH2			+/-				X			X	
GPD1			+							X	X
PSMB4			+		X		X			X	X
FASN			+							X	
RAN			+		X		X				
GDI2			+			X	X				
GDA			+				X				
ACLY			+							X	
FABP4				+/-	X	X	X			X	X
CSRP3				-			X				X
LGALS1				-	X	X					
SELENBP1				+/-	X		X				
DBI				-						X	
MB				-	X		X				X
GOT1				+/-			X			X	X
MCAM				-			X				
APOC1				-						X	X
TMSB4X				+	X		X			X	X
COMP				+			X				
TPM2				-/+			X				
HSP90ab1				+			X			X	
PDIA4				+			X				
MYL1				-/+							X
ESYT1				+			X			X	
