# Supplementary material for: Long-term transcriptomic and proteomic effects in Sprague Dawley rat thyroid and plasma after internal low dose 131I exposure
Source: PLoS One. 2020 Dec 31;15(12):e0244098. doi: 10.1371/journal.pone.0244098 (PMC7774980; doi:10.1371/journal.pone.0244098)
Supplement: S1 Table — (DOCX) [file pone.0244098.s001.docx]

| **A) Uniquely regulated transcripts** | **0.5 kBq**  8 transcripts  7 genes | | **5 kBq**  130 transcripts  125 genes | | | **50 kBq**  17 transcripts  16 genes | | **500 kBq**  95 transcripts  92 genes |
| --- | --- | --- | --- | --- | --- | --- | --- | --- |
|  | |  | |  |  | |  | |
|  | *Calca (-1.81)* | | *Adam7* (1.17) | | | *Acan* (2.55) | | *Acot5* (-1.92) |
|  | *Calca (-1.19)* | | *Adra2a* (1.72) | | | *Adra1b* (-1.91) | | *Agr3* (-2.04) |
|  | *Cxcl10 (1.42)* | | *Aim1l* (-1.98) | | | *Ceacam16* (-1.34) | | *Aldh1a7* (-3.75) |
|  | *Klk1 (-1.93)* | | *Alox12b* (-1.86) | | | *Chad* (2.52) | | *Aldh3a1* (-1.53) |
|  | *LOC100362109 (2.91)* | | *Ankrd22* (-2.14) | | | *Col10a1* (4.54) | | *Alox12e* (1.72) |
|  | *Otog (3.60)* | | *Ano4* (1.20) | | | *Col10a1* (4.61) | | *Aqp3* (-3.50) |
|  | *Sftpa1 (-2.15)* | | *Atp6v0a4* (1.52) | | | *Col2a1* (1.81) | | *Atg9b* (-2.06) |
|  | *Snhg11 (-1.85)* | | *Avp* (-1.45) | | | *Col9a2* (1.47) | | *Atp4b* (-1.48) |
|  |  | | *B3galt1* (-1.14) | | | *Entpd2* (-1.72) | | *Bpifb2* (-2.12) |
|  |  | | *Bhlhe41* (-1.65) | | | *Fam65c* (-1.78) | | *Bpifb6* (-2.04) |
|  |  | | *Cacna1i* (-1.36) | | | *Gdf15* (-1.61) | | *Cabp7* (-1.80) |
|  |  | | *Capns2* (-2.4) | | | *Glis1* (-1.18) | | *Cbln4* (-2.08) |
|  |  | | *Car4* (-1.08) | | | *LOC102546809* (1.30) | | *Chgb* (-2.21) |
|  |  | | *Casq2* (-1.83) | | | Matn1 (2.78) | | *Crtac1* (-2.22) |
|  |  | | *Cldn23* (1.11) | | | Muc16 (2.63) | | *Ctxn3* (-1.69) |
|  |  | | *Colq* (-1.35) | | | *RGD1305045* (-2.56) | | *Cyp2f4* (-2.81) |
|  |  | | *Creld2* (1.36) | | | *Scgb1a1* (2.12) | | *Cyp2t1* (-1.68) |
|  |  | | *Cst6* (-1.17) | | | *Scgb3a1* (2.77) | | *Dcx* (-1.60) |
|  |  | | *Ctgf* (1.22) | | |  | | *Ddc* (-1.21) |
|  |  | | *Cyp2s1* (-1.86) | | |  | | *Dlk2* (-2.30) |
|  |  | | *Dcdc2* (1.06) | | |  | | *Dll3* (-1.98) |
|  |  | | *Degs2* (-3.10) | | |  | | *Dmrt3* (1.72) |
|  |  | | *Derl3* (1.82) | | |  | | *Elmod1* (-2.65) |
|  |  | | *Dio2* (-2.22) | | |  | | *Elovl6* (-1.60) |
|  |  | | *Dsc2* (1.18) | | |  | | *Ephx3* (-1.51) |
|  |  | | *Dsg1* (-2.73) | | |  | | *Fam57a* (-1.65) |
|  |  | | *Fabp5* (-1.55) | | |  | | *Fam83d* (-2.45) |
|  |  | | *Fam111a* (1.47) | | |  | | *Foxa2* (-2.78) |
|  |  | | *Fam20c* (0.96) | | |  | | *Foxl2* (1.91) |
|  |  | | *Fam89a* (-1.13) | | |  | | *Fut1* (-2.94) |
|  |  | | *Fcgr2b* (0.87) | | |  | | *Fxyd3* (-2.78) |
|  |  | | *Fhl1*(-0.93) | | |  | | *Fxyd3* (-2.67) |
|  |  | | *Frmd3* (-1.78) | | |  | | *Galnt13* (2.06) |
|  |  | | *Fxyd6* (-1.6) | | |  | | *Gata3* (1.52) |
|  |  | | *Gbx2* (-2.7) | | |  | | *Gcm2* (1.85) |
|  |  | | *Gk* (-1.52) | | |  | | *Gdap1l1* (-1.72) |
|  |  | | *Grid1*(-1.82) | | |  | | *Gng13* (-1.51) |
|  |  | | *Grk1* (-2.15) | | |  | | *Gpr87* (-2.74) |
|  |  | | *Grk1* (-1.62) | | |  | | *Gsta2* (1.98) |
|  |  | | *Gsdma* (-2.64) | | |  | | *Gzmbl3* (2.63) |
|  |  | | *Gucy2g* (-1.04) | | |  | | *Hoxb7* (-1.59) |
|  |  | | *Hp* (1.38) | | |  | | *Hoxb8* (-2.49) |
|  |  | | *Hsp90b1* (1.48) | | |  | | *Hoxc6* (-2.04) |
|  |  | | *Hspa1b* (-1.51) | | |  | | *Hs3st3a1* (-1.93) |
|  |  | | *Il5* (1.26) | | |  | | *Hs3st6* (-2.21) |
|  |  | | *Irf4* (-2.19) | | |  | | *Hs3st6* (-2.09) |
|  |  | | *Isyna1* (0.96) | | |  | | *Hsbp1l1* (-1.87) |
|  |  | | *Kcng2* (-1.06) | | |  | | *Il36b* (-2.61) |
|  |  | | *Kctd14* (1.00) | | |  | | *Ina* (-2.65) |
|  |  | | *Kirrel3* (1.36) | | |  | | *Insc* (-1.50) |
|  |  | | *Klk10* (-2.74) | | |  | | *Jph3* (-1.32) |
|  |  | | *Klk8* (-3.10) | | |  | | *Kcnb2* (-1.51) |
|  |  | | *Klk9* (-2.31) | | |  | | *Kcnk12* (-1.56) |
|  |  | | *Ky* (-1.00) | | |  | | *Kcnk16* (-1.86) |
|  |  | | *Lmod3* (-0.99) | | |  | | *Kcnmb2* (-1.31) |
|  |  | | *LOC100360165* (2.44) | | |  | | *Krt15* (-3.39) |
|  |  | | *LOC102547811* (-1.55) | | |  | | *Krt17* (-3.00) |
|  |  | | *LOC102547811* (-1.48) | | |  | | *Krt23* (-2.84) |
|  |  | | *LOC102547963* (-2.35) | | |  | | *Krt5* (-2.26) |
|  |  | | *LOC102551188* (-1.51) | | |  | | *Krt7* (-1.74) |
|  |  | | *LOC102551188* (-1.40) | | |  | | *Krt7* (-1.56) |
|  |  | | *LOC102555146* (-1.51) | | |  | | *Lgals7* (-4.27) |
|  |  | | *LOC360919* (-1.68) | | |  | | *LOC100125384* (-2.06) |
|  |  | | *LOC363324* (-1.28) | | |  | | *LOC290595* (-2.05) |
|  |  | | *LOC367516* (-1.51) | | |  | | *LOC681458* (-1.63) |
|  |  | | *LOC686143* (-2.49) | | |  | | *LOC683963* (-1.99) |
|  |  | | *Lonrf3* (1.38) | | |  | | *Lrrc74* (2.71) |
|  |  | | *Mall* (-1.2) | | |  | | *Lrrn2* (1.52) |
|  |  | | *Mfsd10* (1.37) | | |  | | *Magel2* (-3.15) |
|  |  | | *Mnx1* (1.34) | | |  | | *Mdga2* (1.34) |
|  |  | | *Muc20* (0.82) | | |  | | *Pcsk2* (-2.16) |
|  |  | | *Mybph* (-2.47) | | |  | | *Pcyt1b* (1.75) |
|  |  | | *Myh15* (1.75) | | |  | | *Pnoc* (-2.30) |
|  |  | | *Myh6* (-1.19) | | |  | | *Ppp1r17* (-2.04) |
|  |  | | *Myl3* (-2.10) | | |  | | *Rasal1* (1.52) |
|  |  | | *Myl4* (-2.02) | | |  | | *Rhcg* (2.38) |
|  |  | | *Myoz2* (-1.36) | | |  | | *Rup2* (-1.84) |
|  |  | | *Nek10* (-1.08) | | |  | | *Scgn* (-2.43) |
|  |  | | *Nkpd1* (-1.48) | | |  | | *Sdc1* (-1.56) |
|  |  | | *Nkx2* (-10.95) | | |  | | *Serpinb10* (-3.33) |
|  |  | | *Nr1d1* (-1.29) | | |  | | *Serpinb8* (-2.06) |
|  |  | | *Nr4a3* (-1.89) | | |  | | *Slc15a1* (-2.39) |
|  |  | | *Nrarp* (-1.30) | | |  | | *Slc29a4* (-1.78) |
|  |  | | *Ooep* (-2.09) | | |  | | *Slc38a5* (-1.14) |
|  |  | | *Pianp* (2.26) | | |  | | *Slurp1* (-3.93) |
|  |  | | *Pitx1* (-1.05) | | |  | | *Sprr1a* (-3.10) |
|  |  | | *Plk3* (-1.79) | | |  | | *St18* (-1.30) |
|  |  | | *Prr15l* (1.37) | | |  | | *St8sia3* (-1.67) |
|  |  | | *Psors1c2* (-1.80) | | |  | | *Sult4a1* (-1.70) |
|  |  | | *Rasl11b* (1.45) | | |  | | *Sycp2l* (2.02) |
|  |  | | *Rfx4* (-1.47) | | |  | | *Syt4* (-2.33) |
|  |  | | *Rgd1310049* (-0.86) | | |  | | *Them5* (-1.66) |
|  |  | | *RGD1311874* (-1.38) | | |  | | *Tnmd* (1.63) |
|  |  | | *RGD1561909* (-1.96) | | |  | | *Tnr* (-1.75) |
|  |  | | *RGD1562550*(1.66) | | |  | | *Vwa5b2* (-1.75) |
|  |  | | *RGD1562660* (-1.43) | | |  | |  |
|  |  | | *RGD1563060* (-3.51) | | |  | |  |
|  |  | | *RGD1564482* (1.91) | | |  | |  |
|  |  | | *Rprm* (1.29) | | |  | |  |
|  |  | | *RT1-CE10* (-3.16) | | |  | |  |
|  |  | | *RT1-CE16* (1.37) | | |  | |  |
|  |  | | *RT1-CE3* (1.35) | | |  | |  |
|  |  | | *S100a11* (1.01) | | |  | |  |
|  |  | | *Sbk2* (-2.10) | | |  | |  |
|  |  | | *Sbk2* (-1.73) | | |  | |  |
|  |  | | *Sbsn* (-3.75) | | |  | |  |
|  |  | | *Scd1* (-0.80) | | |  | |  |
|  |  | | *Scn1a* (-1.53) | | |  | |  |
|  |  | | *Sdf2l1* (1.33) | | |  | |  |
|  |  | | *Serpinb11* (-2.62) | | |  | |  |
|  |  | | *Serpinb9* (0.80) | | |  | |  |
|  |  | | *Slc37a2* (-0.89) | | |  | |  |
|  |  | | *Slc7a10* (-1.14) | | |  | |  |
|  |  | | *Sst* (3.09) | | |  | |  |
|  |  | | *Tgm3* (-3.03) | | |  | |  |
|  |  | | *Timp3* (0.82) | | |  | |  |
|  |  | | *Tmem213* (1.93) | | |  | |  |
|  |  | | *Tmem213* (2.11) | | |  | |  |
|  |  | | *Tmprss11d* (-2.79) | | |  | |  |
|  |  | | *Tmprss3* (1.44) | | |  | |  |
|  |  | | *Tnni1* (-1.57) | | |  | |  |
|  |  | | *Tnnt1* (-1.09) | | |  | |  |
|  |  | | *Tob1* (-1.08) | | |  | |  |
|  |  | | *Tp63* (-1.78) | | |  | |  |
|  |  | | *Trex2* (-3.25) | | |  | |  |
|  |  | | *Ttc25* (-1.17) | | |  | |  |
|  |  | | *Ubd* (1.11) | | |  | |  |
|  |  | | *Wbscr27* (1.22) | | |  | |  |
|  |  | | *Wnt16* (-1.57) | | |  | |  |
|  |  | | *Vtcn1* (-1.71) | | |  | |  |

| **B) Uniquely regulated thyroid proteins** | **0.5 kBq**  54 proteins | **5 kBq**  112 proteins | **50 kBq**  47 proteins | **500 kBq**  34 proteins |
| --- | --- | --- | --- | --- |
|  | ANP32A (-1.54) | ACTC1 (-1.73) | ACAT1 (-1.57) | ADI1 (1.69) |
|  | BCL2 (2.01) | ADCK3 (-1.96) | ACBD5 (1.59) | AK2 (-2.18) |
|  | BIN2 (1.50) | ADK (-1.52) | ACTB (1.73) | ANPEP (-1.52) |
|  | CCDC43 (1.81) | AK1 (-2.02) | AKR1B1 (-1.50) | ARL3 (-1.73) |
|  | CD48 (-1.50) | ANK3 (-1.50) | APOA1 (1.93) | BAG6 (1.58) |
|  | CEBPA (2.04) | AP2A2 (-1.95) | ATP5L (1.60) | BZW2 (1.64) |
|  | CHGA (1.74) | ARF4 (-2.39) | CABP1 (-1.64) | CALCA (-1.78) |
|  | CPA3 (-1.54) | ARPP19 (-2.08) | CD59 (-1.55) | CAPN2 (-1.76) |
|  | DPEP1 (1.76) | ATG3 (-1.95) | DAB2 (1.68) | CC2D1B (1.56) |
|  | DYNLL1 (-1.79) | ATP5D (-1.58) | ECHDC1 (1.60) | CCDC22 (-1.59) |
|  | EDF1 (-1.59) | ATP5J (1.63) | ECHS1 (1.82) | CD44 (-1.56) |
|  | EIF4A2 (-4.48) | BANF1 (-1.53) | EMB (-1.61) | CHP1 (1.56) |
|  | FAM129B (-1.58) | BANF1 (-1.67) | ENSA (1.70) | CKMT1 (-1.55) |
|  | FHL1 (4.20) | BIN1 (1.64) | F3 (1.63) | CYB5B (-1.54) |
|  | GGH (1.58) | BOD1 (1.53) | FH (1.70) | DBI (1.83) |
|  | GPX3 (1.67) | CACNA1S (-1.65) | GABARAP (1.53) | DLGAP4 (-1.52) |
|  | H1F0 (1.61) | CACNA2D1 (-1.90) | GDA (1.88) | EIF3B (1.55) |
|  | HIST1H1B (-1.67) | CADM4 (-2.58) | GPD1 (-2.47) | EIF5A (-1.64) |
|  | HMBS (-1.77) | CALM1 (1.52) | HDGFRP3 (-1.64) | FLNC (-1.54) |
|  | HN1 (1.74) | CCDC90B (1.53) | HMGB1 (-1.61) | FSTL1 (-1.54) |
|  | HSPA2 (-1.55) | CISD1 (-1.70) | HMGN2 (-2.06) | GADD45GIP1 (1.64) |
|  | ITFG3 (-1.56) | CKMT2 (1.96) | HOMER1 (-1.51) | IGFBP5 (-1.60) |
|  | ITGB1 (-1.64) | COPS3 (-2.38) | HRSP12 (-1.73) | ILF2 (1.54) |
|  | KPNB1 (-1.60) | COQ7 (-1.64) | HSD17B10 (-1.51) | MRPL40 (1.96) |
|  | LRRFIP1 (1.57) | CRYAB (1.76) | IDH3B (-1.67) | MYBPC1 (-1.54) |
|  | MAP1B (1.54) | CST3 (-1.58) | LYPLA1 (-1.51) | NT5E (-1.51) |
|  | MAP2K5 (2.78) | DCN (-1.75) | MAP6 (1.59) | OPTN (2.93) |
|  | MAPK14 (1.77) | DDAH1 (-1.50) | MAPK1 (-1.66) | PCNP (-1.54) |
|  | MYH11 (1.59) | DNAJB11 (-1.87) | MDH1 (-1.79) | RAB1A (-1.85) |
|  | NDUFA9 (1.52) | EIF4EBP1 (1.84) | MDH2 (1.64) | RBM8A (1.55) |
|  | NENF (-1.56) | EMCN (-1.66) | MINPP1 (1.57) | SDPR (1.73) |
|  | NIT2 (2.23) | F10 (-1.77) | MTCO2 (1.53) | TIMM13 (-1.54) |
|  | NUCB2 (1.59) | FDX1 (-1.68) | MTDH (-1.61) | TMSB4X (1.66) |
|  | PABPC1 (1.55) | FNBP1L (-1.54) | NDUFA10 (1.62) | UQCRH (-1.75) |
|  | PDAP1 (2.12) | FXN (-1.52) | NDUFS1 (-1.73) |  |
|  | PDCD4 (-1.51) | GLO1 (-1.58) | NOLC1 (1.66) |  |
|  | PSAP (-1.82) | GLTP (-1.59) | OPA1 (1.56) |  |
|  | PTGES3 (1.55) | GOLGA5 (-1.54) | PRRC1 (-1.62) |  |
|  | RAB7A (1.60) | GRN (-1.81) | PSMB6 (1.75) |  |
|  | RABEP2 (-1.59) | GRPEL1 (-1.82) | PSMD11 (1.63) |  |
|  | RPL19 (1.66) | GSTA4 (-1.55) | RPL22 (1.66) |  |
|  | RPL31 (-1.52) | GSTA5 (2.06) | RPL38 (2.04) |  |
|  | RPL35 (-1.64) | HDLBP (-1.83) | SOD2 (1.60) |  |
|  | RPS25 (-1.56) | HSP90B1 (-1.61) | TMEM126A (-1.68) |  |
|  | RT1-AW2 (-1.53) | HSPA5 (-1.80) | TUBA4A (-1.51) |  |
|  | RUFY3 (1.87) | HSPB1 (-1.75) | UQCRC1 (-1.75) |  |
|  | SDC2 (1.55) | HSPB2 (-1.51) | ZC3H15 (-1.65) |  |
|  | SDF4 (-1.55) | HSPB6 (-1.55) |  |  |
|  | SH3KBP1 (-1.63) | HSPB7 (-1.54) |  |  |
|  | SOD1 (-1.59) | IAH1 (-1.57) |  |  |
|  | SPTAN1 (1.62) | IDH3A (-1.53) |  |  |
|  | TIMM10 (-1.61) | ITGB4 (-1.65) |  |  |
|  | TPM4 (-1.57) | JUP (-2.25) |  |  |
|  | VIMP (2.16) | KRT17 (-1.89) |  |  |
|  |  | KRT8 (-1.51) |  |  |
|  |  | LSAMP (-1.53) |  |  |
|  |  | LUM (-1.62) |  |  |
|  |  | MACROD1 (1.79) |  |  |
|  |  | MACROD1 (1.55) |  |  |
|  |  | MAPT (-1.55) |  |  |
|  |  | MBP (1.62) |  |  |
|  |  | MCCC2 (1.54) |  |  |
|  |  | MPI (-1.51) |  |  |
|  |  | MURC (-1.91) |  |  |
|  |  | MX2 (-1.61) |  |  |
|  |  | MYH7 (-2.51) |  |  |
|  |  | MYH9 (-1.70) |  |  |
|  |  | NDUFS4 (1.60) |  |  |
|  |  | NDUFV2 (1.73) |  |  |
|  |  | NEFH (-1.65) |  |  |
|  |  | NEFL (-1.74) |  |  |
|  |  | NEFM (2.00) |  |  |
|  |  | NUP35 (-1.64) |  |  |
|  |  | OAT (-1.89) |  |  |
|  |  | PACSIN1 (1.58) |  |  |
|  |  | PAM (-1.81) |  |  |
|  |  | PEBP1 (-1.71) |  |  |
|  |  | PFDN2 (-1.96) |  |  |
|  |  | PGRMC2 (-1.76) |  |  |
|  |  | PKIA (1.55) |  |  |
|  |  | PLIN1 (1.92) |  |  |
|  |  | PLVAP (-1.56) |  |  |
|  |  | PNPO (1.84) |  |  |
|  |  | PPIF (1.50) |  |  |
|  |  | PPM1B (-1.68) |  |  |
|  |  | PPP1R2 (-1.54) |  |  |
|  |  | PRDX1 (-1.58) |  |  |
|  |  | PRX (-1.63) |  |  |
|  |  | RAD23B (-1.63) |  |  |
|  |  | RPL12 (-1.51) |  |  |
|  |  | RPL7A (-1.51) |  |  |
|  |  | RPN1 (1.87) |  |  |
|  |  | RWDD4 (-1.78) |  |  |
|  |  | SEC31A (-1.52) |  |  |
|  |  | SGTA (1.54) |  |  |
|  |  | SNCA (1.65) |  |  |
|  |  | SSR4 (-1.52) |  |  |
|  |  | STMN1 (-1.57) |  |  |
|  |  | SULT1A1 (-3.27) |  |  |
|  |  | SYNPO (-1.60) |  |  |
|  |  | TALDO1 (-1.60) |  |  |
|  |  | TIMM8B (-1.66) |  |  |
|  |  | T-kinnogen 2 (1.52) |  |  |
|  |  | TMEM109 (-1.80) |  |  |
|  |  | TNNT1 (-1.51) |  |  |
|  |  | TXNDC15 (-1.51) |  |  |
|  |  | UBA1 (1.56) |  |  |
|  |  | UBAC1 (-1.70) |  |  |
|  |  | UBE2V2 (1.55) |  |  |
|  |  | UBXN4 (-1.67) |  |  |
|  |  | VAPB (1.63) |  |  |
|  |  | YWHAG (-1.61) |  |  |

| **C) Uniquely regulated plasma proteins** | **0.5 kBq**  47 proteins | **5 kBq**  19 proteins | **50 kBq**  50 proteins | **500 kBq**  48 proteins |
| --- | --- | --- | --- | --- |
|  | A2M (2.97) | ALPI (2.49) | ACLY (1.55) | AKR1B1 (-1.60) |
|  | ACO2 (1.50) | ANXA1 (-1.63) | ACP1 (2.45) | ANXA5 (-1.78) |
|  | ANXA2 (-1.78) | CELSR3 (-1.63) | AKR7A2 (1.53) | APOC1 (1.89) |
|  | APRT (-1.61) | CSAD (-1.51) | ALDOB (1.85) | CALR (-1.94) |
|  | ATP6V0C (1.66) | DPP7 (-1.83) | ATIC (-1.58) | CNTN1 (-1.83) |
|  | C4BPA (-1.58) | ENO3 (-1.84) | ATP5A1 (1.55) | CNTN2 (1.50) |
|  | C4BPB (2.31) | F9 (2.07) | AUNIP (1.93) | CSF1R (-1.62) |
|  | CALD1 (-3.56) | GSTA3 (-1.53) | BLVRA (1.78) | DBI (-1.61) |
|  | CAP1 (3.00) | GSTA3 (1.52) | CAND1 (1.87) | DDT (-1.86) |
|  | CCS (-1.70) | GSTM2 (-1.59) | CARHSP1 (-1.60) | DPEP2 (1.53) |
|  | CFL1 (1.63) | HSPE1 (1.52) | CAT (2.79) | EPHX1 (-1.58) |
|  | CKMT2 (-1.63) | IDH1 (1.54) | CHI3L1 (1.52) | FABP3 (-1.67) |
|  | CLEC11A (2.28) | LPP (1.58) | CPS1 (1.89) | FABP4 (-1.51) |
|  | CORO1A (2.32) | MAPRE1 (-1.61) | CTSL (1.55) | GOT1 (-1.77) |
|  | DHTKD1 (-1.55) | NUCB1 (-1.70) | DAK (1.81) | HIST1H1E (1.54) |
|  | FBP1 (1.55) | OSMR (2.47) | EIF1A (2.26) | HSP90AB1 (-1.53) |
|  | GGT5 (1.73) | PLBD2 (1.61) | ESD (-1.57) | HYAL1 (-1.54) |
|  | GNAI3 (1.54) | RTN4 (1.56) | FAM129A (2.02) | IGFBP6 (1.54) |
|  | GRIN2D (4.17) | STAT5B (-1.51) | FASN (1.55) | KALRN (1.65) |
|  | HINT1 (-1.75) |  | FCN2 (1.56) | LGALS1 (-1.61) |
|  | HIST1H4B (1.70) |  | GDA (1.73) | LTBP1 (1.61) |
|  | IDH2 (-1.68) |  | GDI2 (1.54) | MAP2K5 (-1.58) |
|  | ILK (-1.53) |  | GLO1 (2.29) | MAPK14 (1.75) |
|  | L1CAM (1.73) |  | GPD1 (1.52) | MB (1.70) |
|  | LDHB (-1.63) |  | GPX1 (1.73) | MCAM (1.61) |
|  | MANF (1.66) |  | GSTA4 (1.78) | MYL1 (1.57) |
|  | MAPRE2 (-1.56) |  | Histone H2A type 3 (-1.70) | NBL1 (-2.13) |
|  | MYL3 (-1.54) |  | HPSE (1.50) | NF1 (-1.51) |
|  | MYL9 (-1.83) |  | IMPA1 (1.59) | PARK7 (-1.69) |
|  | NUDC (1.89) |  | KIF2C (1.51) | PDIA4 (-2.63) |
|  | PAM (1.55) |  | LAP3 (1.61) | PEBP1 (1.94) |
|  | PFN1 (-1.75) |  | NIT1 (1.53) | PECAM1 (-1.83) |
|  | PROS1 (-2.39) |  | PDE5A (1.53) | PODXL (1.79) |
|  | RAB27B (1.96) |  | PDLIM1 (-1.52) | PRKAR2B (-1.55) |
|  | RAB7A (1.98) |  | PKLR (-1.71) | PSMB9 (2.51) |
|  | RAC1 (-1.64) |  | PLEC (1.53) | PTGIS (-1.63) |
|  | RGN (-1.77) |  | PLEK (-1.67) | RHD (-1.55) |
|  | RT1-AW2 (-1.54) |  | PPP2R1B (1.50) | RNH1 (-1.59) |
|  | SEPT7 (-1.55) |  | PRDX5 (2.00) | SELENBP1 (-1.55) |
|  | SLC2A3 (-1.62) |  | PSMB1 (1.73) | SERPINA1 (-2.47) |
|  | SLC9A3R1 (-1.62) |  | PSMB4 (1.68) | SERPINA7 (1.80) |
|  | SNED1 (1.96) |  | RAN (1.51) | SIAE (1.52) |
|  | SRC (-1.79) |  | RGS18 (1.67) | SNCG (-1.87) |
|  | TES (-1.68) |  | S100A8 (1.54) | SND1 (-1.63) |
|  | TGM1 (1.58) |  | SERPINE2 (1.80) | SOD1 (- 2.05) |
|  | TMOD2 (1.85) |  | SHBG (2.36) | TPM2 (-1.58) |
|  | YWHAB (-1.51) |  | SKAP2 (-1.60) | TRIM9 (-1.59) |
|  |  |  | SLC4A1 (1.88) | TUBA1C (1.74) |
|  |  |  | UBB (2.18) |  |
|  |  |  | UBE2N (1.75) |  |
