# Supplementary material for: Long-term transcriptomic and proteomic effects in Sprague Dawley rat thyroid and plasma after internal low dose 131I exposure
Source: PLoS One. 2020 Dec 31;15(12):e0244098. doi: 10.1371/journal.pone.0244098 (PMC7774980; doi:10.1371/journal.pone.0244098)
Supplement: S2 Table — (DOCX) [file pone.0244098.s002.docx]

.

| **Commonly regulated transcripts and proteins three test groups** | **0.5 kBq** | **5 kBq** | **50 kBq** | **500 kBq** |
| --- | --- | --- | --- | --- |
| **Thyroid transcripts** |  |  |  |  |
| **0.5, 5 and 500 KBq** |  |  |  |  |
| *Vegfb* | 4.43 | 5.74 |  | 8.34 |
| **5, 50 and 500 kBq** |  |  |  |  |
| *Ankrd2* |  | -2.09 | -2.17 | -2.13 |
| **Thyroid proteins** |  |  |  |  |
| **0.5, 5 and 50 kBq** |  |  |  |  |
| RBM20 | -1.86 | -2.37 | -1.52 |  |
| APOD | -1.84 | -3.47 | -2.45 |  |
| AGMN | -2.76 | -1.93 | -1.53 |  |
| GSTZ1 | 1.56 | 1.69 | 1.56 |  |
| HIST1H4b | 3.19 | 2.40 | -2.18 |  |
| Histone H2B type 1 | 4.29 | 1.78 | -3.49 |  |
| CAPZA2 | 1.60 | 1.67 | 1.76 |  |
| Histone H2A type 3 | 3.04 | 1.90 | -1.52 |  |
| FAM213A | 1.71 | 1.53 | 2.66 |  |
| **0.5, 5 and 500 KBq** |  |  |  |  |
| A1M | 1.66 | 1.89 |  | 1.70 |
| AIFM1 | 2.47 | 2.38 |  | 2.04 |
| AKR7A2 | 1.84 | 1.80 |  | 1.75 |
| ARHGDIA | 1.66 | 1.79 |  | 1.67 |
| ATP5H | -1.51 | -2.48 |  | -1.50 |
| BASP1 | -1.64 | -1.64 |  | -1.56 |
| BCKDHA | 1.90 | 1.93 |  | 2.06 |
| BSG | -1.76 | -2.13 |  | -1.99 |
| C3 | 2.90 | 3.12 |  | 2.44 |
| CA1 | 2.29 | 1.73 |  | 1.65 |
| CA2 | 3.18 | 2.40 |  | 1.92 |
| CAT | 1.74 | 2.38 |  | 1.80 |
| CES1D | 2.18 | 3.29 |  | 3.04 |
| CLIC5 | -1.73 | -1.62 |  | -1.84 |
| COL1A1 | 1.97 | 2.27 |  | 1.71 |
| COL1A2 | 1.83 | 2.06 |  | 2.08 |
| COQ9 | -1.63 | -2.57 |  | -1.55 |
| COX7A2 | -1.55 | -2.04 |  | -2.06 |
| CP | 1.89 | 1.99 |  | 1.79 |
| CRELD1 | -1.52 | -2.00 |  | -1.61 |
| CTSD | 1.62 | 1.90 |  | 1.79 |
| CWC15 | -1.54 | -1.69 |  | -1.79 |
| CYB5R1 | 2.07 | 1.90 |  | 1.64 |
| DDX39B | 1.53 | 1.90 |  | 1.55 |
| EEF1A1 | 3.24 | 3.43 |  | 3.07 |
| EIF3G | 1.53 | 1.65 |  | 1.58 |
| ESYT1 | 1.54 | 2.04 |  | 1.70 |
| ETFDH | 1.82 | 1.52 |  | 1.84 |
| FGB | 2.00 | 1.99 |  | 1.84 |
| FXYD1 | -1.78 | -2.55 |  | -1.86 |
| GAA | 2.61 | 3.19 |  | 2.35 |
| GAP43 | -2.05 | -2.10 |  | -2.02 |
| GMPR | 1.97 | 1.64 |  | 1.62 |
| GNA13 | -1.77 | -1.57 |  | -1.54 |
| GNB2L1 | 1.99 | 2.39 |  | 2.07 |
| GPC1 | -1.72 | -2.26 |  | -1.64 |
| GSN | 1.62 | 1.73 |  | 1.77 |
| GSTP1 | 1.51 | 2.44 |  | 2.46 |
| H3F3B | 4.07 | 3.11 |  | 1.86 |
| HNRNPA2B1 | 1.57 | 1.87 |  | 1.65 |
| HSP90Ab1 | 1.50 | 1.50 |  | 1.58 |
| Ig kappa chain C region A allele | 1.71 | 1.82 |  | 1.50 |
| IGG-2A | 2.26 | 2.41 |  | 1.65 |
| IGH-1A | 3.15 | 2.98 |  | 2.30 |
| LAP3 | 1.66 | 1.99 |  | 1.66 |
| LHPP | 1.63 | 1.53 |  | 1.87 |
| MCAM | -1.50 | -1.56 |  | -1.62 |
| MCM8 | -1.59 | -2.29 |  | -1.58 |
| MRRF | -1.56 | -2.10 |  | -1.72 |
| MT-ATP8 | -1.61 | -2.06 |  | -1.59 |
| MYL3 | -1.67 | -6.66 |  | -3.82 |
| NDRG2 | -1.70 | -2.16 |  | -1.51 |
| NID2 | 1.69 | 1.68 |  | 1.85 |
| NUDT9 | -1.71 | -1.99 |  | -1.80 |
| NUP93 | -1.64 | -2.71 |  | -2.07 |
| OGDH | 2.35 | 2.29 |  | 2.25 |
| PDLIM2 | -1.82 | -2.41 |  | -2.41 |
| PFKM | 3.54 | 3.06 |  | 2.41 |
| PGD | 1.53 | 1.93 |  | 1.63 |
| PHRF1 | 1.90 | 1.94 |  | 2.64 |
| PLG | 1.95 | 1.83 |  | 1.94 |
| PPIB | 1.75 | 2.25 |  | 1.76 |
| PRDX2 | 1.55 | 1.66 |  | 1.61 |
| PRKCDBP | -1.56 | -1.57 |  | -1.56 |
| PRSS1 | -1.58 | -1.81 |  | -1.85 |
| PSMB2 | 1.55 | 1.72 |  | 1.58 |
| PSPC1 | 1.56 | 2.20 |  | 1.92 |
| PYGB | 2.80 | 2.91 |  | 2.29 |
| RAb7A | 2.04 | 2.49 |  | 1.80 |
| RBMXRTL | 1.89 | 1.94 |  | 1.83 |
| RPL10A | 2.05 | 2.07 |  | 1.67 |
| RPS14 | 2.16 | 2.19 |  | 1.89 |
| RPS15A | 2.86 | 2.72 |  | 2.00 |
| RPS23 | 1.93 | 1.94 |  | 1.69 |
| RPS26 | 2.56 | 2.39 |  | 1.89 |
| RPS3A | 2.00 | 1.83 |  | 1.54 |
| RPS4X | 2.53 | 2.58 |  | 1.89 |
| RPS8 | 4.32 | 3.83 |  | 3.47 |
| S100A10 | -1.82 | -2.01 |  | -2.23 |
| S100A4 | -1.53 | -2.43 |  | -2.08 |
| SCG3 | -2.26 | -1.56 |  | -2.51 |
| SCN4b | -1.50 | -1.93 |  | -1.55 |
| SERPINH1 | 1.76 | 2.13 |  | 2.07 |
| SNCG | -1.69 | -1.87 |  | -2.01 |
| ST13 | -1.50 | -1.72 |  | -1.65 |
| SUCLG1 | 2.15 | 2.00 |  | 1.60 |
| TCP1 | 1.72 | 1.67 |  | 1.59 |
| TF | 1.73 | 1.68 |  | 1.67 |
| TPM1 | -1.72 | -2.32 |  | -1.98 |
| TPO | 1.83 | 2.38 |  | 1.71 |
| TPT1 | -1.62 | -2.12 |  | -1.64 |
| TWF1 | 1.85 | 2.68 |  | 1.90 |
| UCHL1 | -2.10 | -1.68 |  | -1.99 |
| VAMP3 | -1.52 | -1.54 |  | -1.73 |
| VDAC2 | 1.66 | 1.76 |  | 1.70 |
| VDAC3 | 2.31 | 2.26 |  | 2.35 |
| WDR1 | 2.73 | 2.91 |  | 2.32 |
| VWA1 | 1.51 | 1.72 |  | 1.80 |
| **0.5, 50 and 500 kBq** |  |  |  |  |
| ATP5C1 | -1.93 |  | -1.70 | -1.63 |
| CAPZB | 8.92 |  | 14.22 | -1.82 |
| CPT2 | 2.05 |  | 1.61 | 1.51 |
| GOT1 | 1.68 |  | 1.72 | 1.77 |
| Ig kappa chain C region B allel | 1.92 |  | 1.76 | 1.62 |
| KRT19 | 1.75 |  | 2.18 | 1.78 |
| PTH | 1.52 |  | 1.59 | 1.54 |
| TUBA8 | 1.50 |  | 1.62 | 1.58 |
| WASL | 1.74 |  | 1.97 | 1.68 |
| **5, 50 and 500 kBq** |  |  |  |  |
| AKR1A1 |  | 1.68 | 1.84 | 1.51 |
| APOA2 |  | -1.53 | -1.80 | -1.56 |
| ARMC10 |  | -1.53 | -1.50 | -1.89 |
| ATP6V1B2 |  | -1.74 | -1.80 | -1.79 |
| CCT2 |  | -2.11 | -2.33 | -1.71 |
| CDNF |  | -1.58 | -1.53 | -1.69 |
| DSTN |  | -1.74 | -1.50 | -1.62 |
| GATC |  | -2.03 | -1.66 | -1.72 |
| HBA1 |  | -1.61 | -1.74 | -1.52 |
| Haemoglobin subunit beta-2 |  | -1.84 | -1.62 | -2.33 |
| HSDL2 |  | -2.27 | -1.52 | -1.53 |
| HSPB3 |  | -1.54 | 1.83 | -1.59 |
| IL1RAP |  | 1.77 | 1.53 | 1.55 |
| IRGC |  | -1.94 | -1.57 | -1.56 |
| KNG1 |  | 1.59 | 1.54 | 1.70 |
| KRT13 |  | 1.76 | 1.51 | 1.64 |
| KRT15 |  | 2.44 | 1.82 | 1.73 |
| LGALS7 |  | -6.23 | 1.63 | -6.60 |
| LXN |  | 2.18 | 1.98 | 2.15 |
| MAPK3 |  | -3.22 | -1.76 | -2.38 |
| MUSTN1 |  | -2.14 | -3.17 | -2.93 |
| NRADD |  | 1.54 | 1.78 | 1.54 |
| PARVA |  | -1.72 | -1.94 | -1.57 |
| PYURF |  | 1.80 | 1.53 | 1.60 |
| RPL18 |  | -1.80 | -1.77 | -1.73 |
| SCGB1A1 |  | -3.39 | 1.55 | -4.04 |
| SERPINA6 |  | -4.47 | 1.52 | -6.15 |
| SEPT8 |  | 2.05 | 1.53 | 1.78 |
| SLC3A2 |  | 1.51 | 1.62 | 1.67 |
| SVIP |  | 1.91 | 1.80 | 1.57 |
| TNNI1 |  | -1.68 | -2.15 | -1.69 |
| UPF0729 protein C18orf32 homolog |  | -2.45 | -1.55 | -2.47 |
| USMG5 |  | 1.69 | 1.66 | 1.67 |
| VAT1 |  | -2.14 | -1.52 | -1.59 |
| VCP |  | -1.92 | -1.65 | -1.92 |
| **Plasma proteins** |  |  |  |  |
| **0.5, 5 and 50 kBq** |  |  |  |  |
| BAAT | -1.69 | -1.73 | 1.50 |  |
| CYP27B1 | 1.68 | 1.55 | 1.57 |  |
| DSG4 | 3.77 | 1.78 | 1.65 |  |
| RRAS | -1.69 | -1.69 | -1.69 |  |
| SERPINB10 | -2.06 | 1.55 | -1.76 |  |
| TAOK3 | 6.01 | 1.55 | 1.78 |  |
| TGM3 | 5.67 | 1.85 | 1.61 |  |
| **0.5, 5 and 500 KBq** |  |  |  |  |
| BASP1 | -1.54 | -2.69 |  | -1.82 |
| MCPT1 | -1.51 | -1.77 |  | -1.57 |
| NOVA1 | 2.02 | 2.00 |  | 3.19 |
| PPP1R7 | 1.84 | 1.63 |  | 1.84 |
| **0.5, 50 and 500 KBq** |  |  |  |  |
| ALDH2 | -1.99 |  | -3.12 | -6.17 |
| CAPN1 | -1.83 |  | -1.85 | 1.61 |
| CSRP3 | 1.53 |  | 1.74 | 1.83 |
| FTH1 | -1.50 |  | -2.5 | -4.95 |
| FTL1 | 1.63 |  | 1.56 | 1.50 |
| LGALS5 | 3.44 |  | 4.31 | 1.53 |
| PRKCA | 1.54 |  | -1.56 | -2.01 |
| PSMB3 | 1.58 |  | -1.98 | -1.91 |
| **5, 50 and 500 kBq** |  |  |  |  |
| PPIF |  | 1.64 | -1.53 | 1.66 |
