# Supplementary material for: Long-term transcriptomic and proteomic effects in Sprague Dawley rat thyroid and plasma after internal low dose 131I exposure
Source: PLoS One. 2020 Dec 31;15(12):e0244098. doi: 10.1371/journal.pone.0244098 (PMC7774980; doi:10.1371/journal.pone.0244098)
Supplement: S3 Table — (DOCX) [file pone.0244098.s003.docx]

| **Commonly regulated transcripts and proteins two test groups** | **0.5 kBq** | **5 kBq** | **50 kBq** | **500 kBq** |
| --- | --- | --- | --- | --- |
| **0.5 and 5 kBq**  **Tyroid transcripts** |  |  |  |  |
| *Klk1c2* | -3.43 | -1.88 |  |  |
| *Klk1c9* | -2.17 | -3.32 |  |  |
| *Myh13* | -3.00 | -3.95 |  |  |
| **Thyroid proteins** |  |  |  |  |
| AKAP12 | -1.86 | -1.62 |  |  |
| APCS | 2.21 | 1.82 |  |  |
| ATPIF1 | 1.59 | 1.67 |  |  |
| B9D2 | 1.58 | 1.50 |  |  |
| BGN | -1.55 | -1.61 |  |  |
| CES1C | 1.66 | 1.83 |  |  |
| CHGB | -3.33 | -2.19 |  |  |
| ECHDC3 | 1.53 | 1.87 |  |  |
| EIF3A | 1.67 | 2.59 |  |  |
| FCER1G | -1.85 | -1.83 |  |  |
| GALNT13 | 1.51 | 2.03 |  |  |
| GLG1 | -1.51 | -1.59 |  |  |
| H2AFZ | 2.65 | 1.94 |  |  |
| HEXB | 1.59 | 1.54 |  |  |
| HIST1H1E | 2.07 | 1.62 |  |  |
| HSPB8 | -1.57 | -2.44 |  |  |
| KRT1 | -1.80 | -3.18 |  |  |
| KRT2 | -1.63 | -3.17 |  |  |
| KRT6A | -1.54 | -2.20 |  |  |
| LGALS5 | 3.56 | 1.63 |  |  |
| LRRC59 | 2.39 | 1.54 |  |  |
| MAP1LC3A | -1.51 | -1.53 |  |  |
| ME1 | 1.76 | 1.78 |  |  |
| NUDT21 | 1.71 | 1.50 |  |  |
| RESP18 | -2.34 | -1.63 |  |  |
| RPL17 | 3.63 | 2.37 |  |  |
| RPL29 | 1.93 | 1.66 |  |  |
| RPS10 | 1.71 | 1.81 |  |  |
| RPS2 | 2.12 | 2.19 |  |  |
| SLC25A3 | 2.09 | 1.57 |  |  |
| STMN1 | 1.77 | 1.52 |  |  |
| TG | 2.04 | 2.66 |  |  |
| TRIP10 | -1.72 | -1.70 |  |  |
| UCP1 | 1.94 | 1.93 |  |  |
| **Plasma proteins** |  |  |  |  |
| ADH1 | -1.70 | -2.17 |  |  |
| CEACAM1 | 2.41 | -1.61 |  |  |
| CRYAB | 2.09 | -1.54 |  |  |
| CTH | -2.56 | -2.20 |  |  |
| FABP1 | -1.79 | -1.66 |  |  |
| GSTA2 | -2.49 | -2.58 |  |  |
| GSTM1 | -2.07 | -1.66 |  |  |
| PAICS | 1.63 | 1.96 |  |  |
| RT1-AW2 | -3.56 | -5.78 |  |  |
| **0.5 and 50 kBq**  **Thyroid proteins** |  |  |  |  |
| CRK | 1.64 |  | 1.51 |  |
| GPNMB | -1.97 |  | 1.55 |  |
| HMGA1 | -1.86 |  | 1.65 |  |
| HMGN2 | -2.47 |  | 1.71 |  |
| JPH2 | -1.53 |  | 2.25 |  |
| NCL | -1.87 |  | 1.58 |  |
| NUCKS1 | -1.68 |  | 1.76 |  |
| OCM | 1.60 |  | 2.24 |  |
| PDHA1 | 1.52 |  | 1.53 |  |
| RPS7 | -1.74 |  | 1.53 |  |
| **Plasma proteins** |  |  |  |  |
| URINARY PROTEIN 1 | -3.33 |  | -1.89 |  |
| ALOX12 | -1.73 |  | -1.90 |  |
| AOX1 | 1.99 |  | 1.77 |  |
| ATP2A3 | -2.09 |  | -2.56 |  |
| BID | -1.83 |  | -2.20 |  |
| BLMH | 1.55 |  | 1.93 |  |
| CA1 | 3.24 |  | 2.00 |  |
| CA2 | 2.80 |  | 1.72 |  |
| CALD1 | -1.92 |  | -2.39 |  |
| CANX | -1.87 |  | -1.77 |  |
| CLIP2 | -1.71 |  | -1.91 |  |
| COTL1 | -1.71 |  | -1.88 |  |
| DLGAP4 | -2.02 |  | -2.51 |  |
| EEF1A1 | 1.57 |  | 1.55 |  |
| FLNC | -1.54 |  | -1.60 |  |
| G6B | -2.92 |  | -2.92 |  |
| GSTP1 | -1.56 |  | -1.61 |  |
| H1F0 | 1.94 |  | 1.63 |  |
| HAGH | 1.99 |  | 1.51 |  |
| HRSP12 | -1.52 |  | -1.97 |  |
| JUP | 2.17 |  | 3.02 |  |
| LMNA | 1.65 |  | 2.33 |  |
| LMNB1 | 1.64 |  | 1.57 |  |
| LYN | -1.97 |  | -1.75 |  |
| MYH9 | -1.53 |  | -1.52 |  |
| NPM1 | 1.64 |  | 3.30 |  |
| PACSIN2 | 1.85 |  | 1.70 |  |
| PEPD | 1.51 |  | 1.53 |  |
| PLCB4 | 1.96 |  | 2.08 |  |
| PPP1R12A | -1.77 |  | -1.57 |  |
| PRDX4 | 2.13 |  | 2.50 |  |
| PROC | 1.80 |  | 1.56 |  |
| PSMA3 | 1.62 |  | 1.64 |  |
| PSMB7 | 1.63 |  | 1.52 |  |
| RAP2B | -2.00 |  | -1.74 |  |
| RPL6 | 2.00 |  | 2.15 |  |
| SELP | -1.73 |  | -1.66 |  |
| SPINK3 | -3.60 |  | -2.34 |  |
| SPP2 | 1.56 |  | 1.79 |  |
| SPR | 2.49 |  | 1.88 |  |
| TAGLN2 | -1.67 |  | -1.91 |  |
| TPM4 | -1.63 |  | -1.79 |  |
| TPP2 | 2.15 |  | 2.03 |  |
| **0.5 and 500 kBq**  **Thyroid transcript** |  |  |  |  |
| *Calcb* | -1.87 |  |  | -2.25 |
| *Celf3* | -1.95 |  |  | -2.57 |
| *Elavl4* | -1.69 |  |  | -2.17 |
| *Hoxb6* | -1.66 |  |  | -1.92 |
| *Scg3* | -1.67 |  |  | -2.09 |
| *Slc7a14* | -1.70 |  |  | -2.29 |
| *Slc7a14* | -1.70 |  |  | -2.16 |
| *Sun5* | -1.94 |  |  | -2.66 |
| **Thyroid proteins** |  |  |  |  |
| BID | -1.51 |  |  | -1.66 |
| CAMK2G | 1.61 |  |  | 1.63 |
| CHGB | -3.33 |  |  | -1.74 |
| CLIP2 | -1.59 |  |  | -1.51 |
| COX6A1 | -1.83 |  |  | -1.80 |
| CPE | -2.20 |  |  | -1.71 |
| CPLX2 | -1.83 |  |  | -1.68 |
| ERC1 | -1.63 |  |  | -1.73 |
| IGFBP4 | -2.44 |  |  | -3.29 |
| LMAN1 | -1.58 |  |  | -1.59 |
| MPZ | 1.90 |  |  | 1.77 |
| PCSK1N | -2.24 |  |  | -2.53 |
| PCSK2 | -1.98 |  |  | -1.97 |
| PRKAR2A | 1.52 |  |  | 1.58 |
| RARS | 1.53 |  |  | 1.63 |
| SCG5 | -2.00 |  |  | -3.08 |
| VAMP2 | -1.52 |  |  | -1.65 |
| VAMP8 | -1.58 |  |  | -1.58 |
| **Plasma proteins** |  |  |  |  |
| BIN2 | -1.72 |  |  | 1.52 |
| COMP | 1.58 |  |  | 1.57 |
| CST3 | -1.57 |  |  | -1.53 |
| DMBT1 | 1.79 |  |  | 2.10 |
| EML1 | -1.56 |  |  | 1.83 |
| GBP2 | 1.73 |  |  | 1.81 |
| GPNMB | 2.36 |  |  | 3.43 |
| MYH4 | 1.67 |  |  | 1.84 |
| PSMA4 | 1.55 |  |  | 1.55 |
| TG | 2.32 |  |  | 3.17 |
| **5 and 50 kBq**  **Thyroid transcripts** |  |  |  |  |
| *Bpifa1* |  | 1.40 | 2.35 |  |
| *Dbp* |  | -2.03 | -1.81 |  |
| *Dbp* |  | -2.06 | -1.81 |  |
| *Dbp* |  | -2.01 | -1.81 |  |
| *LOC171161* |  | 1.87 | 2.34 |  |
|  |  |  |  |  |
|  |  |  |  |  |
| **Thyroid proteins** |  |  |  |  |
| ACAN |  | -1.73 | 1.69 |  |
| CMSS1 |  | -1.71 | -1.74 |  |
| FABP5 |  | -1.64 | 1.76 |  |
| GPT |  | 1.70 | 1.93 |  |
| HMMR |  | -5.86 | 1.85 |  |
| KRAS |  | 1.87 | 1.69 |  |
| NEXN |  | -1.65 | -1.62 |  |
| PBXIP1 |  | -1.86 | -1.55 |  |
| PPP1R1A |  | -1.67 | -1.66 |  |
| PVALB |  | -1.70 | -1.60 |  |
| SEC22B |  | 1.75 | 1.61 |  |
| SIDT2 |  | -1.84 | -1.70 |  |
| SMPX |  | -3.10 | -1.64 |  |
| **Plasma proteins** |  |  |  |  |
| BPIFA2 |  | 2.31 | 1.55 |  |
| GP5 |  | 1.70 | 2.29 |  |
| MDH2 |  | 1.53 | -1.52 |  |
| PRDX2 |  | 1.55 | 1.97 |  |
| SRGN |  | -1.54 | 2.46 |  |
| **5 and 500 kBq**  **Thyroid transripts** |  |  |  |  |
|  |  |  |  |  |
| *Abca12* |  | -2.94 |  | -2.51 |
| *Ada* |  | -2.72 |  | -3.10 |
| *Adtrp* |  | -1.19 |  | -2.29 |
| *Anxa9* |  | -2.27 |  | -2.11 |
| *Aox4* |  | -3.42 |  | -3.49 |
| *Asprv1* |  | -5.81 |  | -5.93 |
| *Asprv1* |  | -3.76 |  | -5.93 |
| *Asprv1* |  | -5.81 |  | -3.91 |
| *Asprv1* |  | -3.76 |  | -3.91 |
| *Bpifa5* |  | -3.96 |  | -3.83 |
| *Calml5* |  | -4.15 |  | -5.88 |
| *Cldn17* |  | -1.74 |  | -2.14 |
| *Cnfn* |  | -2.88 |  | -2.67 |
| *Col17a1* |  | -1.69 |  | -2.27 |
| *Crisp1* |  | -4.25 |  | -4.15 |
| *Cryba4* |  | -1.26 |  | -1.99 |
| *Csrp3* |  | -1.86 |  | -1.77 |
| *Csta* |  | -4.46 |  | -5.18 |
| *Dapl1* |  | -2.16 |  | -2.57 |
| *Defb4* |  | -4.52 |  | -4.38 |
| *Dmkn* |  | -4.19 |  | -2.90 |
| *Fam25a* |  | -3.23 |  | -3.23 |
| *Fam25a* |  | -4.25 |  | -3.23 |
| *Fam25a* |  | -3.23 |  | -4.45 |
| *Fam25a* |  | -4.25 |  | -4.45 |
| *Fetub* |  | -2.49 |  | -3.16 |
| *Gjb2* |  | -1.98 |  | -1.78 |
| *Grhl3* |  | -2.55 |  | -2.47 |
| *Klk10* |  | -2.74 |  | -3.51 |
| *Klk11* |  | -2.62 |  | -3.03 |
| *Klk12* |  | -3.48 |  | -3.22 |
| *Klk13* |  | -2.88 |  | -3.11 |
| *Klk14* |  | -3.00 |  | -2.98 |
| *Kprp* |  | -3.99 |  | -4.06 |
| *Krt13* |  | -3.71 |  | -4.70 |
| *Krt4* |  | -3.92 |  | -3.90 |
| *Krt78* |  | -4.57 |  | -4.76 |
| *Krt78* |  | -3.23 |  | -4.76 |
| *Krt78* |  | -3.64 |  | -4.76 |
| *Krt78* |  | -4.57 |  | -4.12 |
| *KRrt8* |  | -3.23 |  | -4.12 |
| *Krt78* |  | -3.64 |  | -4.12 |
| *Krt78* |  | -4.57 |  | -3.26 |
| *Krt78* |  | -3.23 |  | -3.26 |
| *Krt78* |  | -3.64 |  | -3.26 |
| *Lce1f* |  | -2.91 |  | -3.38 |
| *Lce1l* |  | -2.89 |  | -2.94 |
| *Lce1m* |  | -2.30 |  | -2.01 |
| *LOC100910442* |  | -1.40 |  | -1.35 |
| *LOC102546758* |  | -3.20 |  | -3.38 |
| *LOC102551453* |  | -2.03 |  | -3.10 |
| *LOC102552128* |  | -4.51 |  | -4.51 |
| *LOC102552326* |  | -4.06 |  | -4.12 |
| *LOC102557206* |  | -1.46 |  | -1.55 |
| *LOC102557467* |  | -1.45 |  | -1.55 |
| *LOC298795* |  | -1.51 |  | -1.90 |
| *LOC363337* |  | -1.83 |  | -1.41 |
| *LOC363337* |  | -1.29 |  | -1.41 |
| *LOC363337* |  | -1.49 |  | -1.41 |
| *LOC363337* |  | -1.52 |  | -1.41 |
| *LOC679711* |  | -1.50 |  | -1.48 |
| *LOC685351* |  | -2.29 |  | -2.60 |
| *Lor* |  | -4.37 |  | -4.65 |
| *Ly6d* |  | -3.84 |  | -4.33 |
| *LY6G6C* |  | -1.98 |  | -1.82 |
| *Mt4* |  | -5.03 |  | -5.43 |
| *Ocm2* |  | -2.97 |  | -2.83 |
| *Pglyrp3* |  | -2.72 |  | -2.46 |
| *Pglyrp3b* |  | -3.32 |  | -2.29 |
| *Pkp1* |  | -2.65 |  | -3.50 |
| *Psapl1* |  | -3.11 |  | -4.14 |
| *RGD1310935* |  | -3.94 |  | -4.24 |
| *RGD1560559* |  | -4.86 |  | -4.91 |
| *RGD1562234* |  | -4.28 |  | -4.15 |
| *RGD1562885* |  | -3.71 |  | -4.42 |
| *RGD1564972* |  | -2.93 |  | -2.97 |
| *RGD1566380* |  | -2.76 |  | -1.59 |
| *Rnase2* |  | 1.59 |  | 1.71 |
| *Rprml* |  | -1.52 |  | -1.66 |
| *Rptn* |  | -3.70 |  | -3.81 |
| *RT1-CE15* |  | 1.43 |  | 1.01 |
| *S100a14* |  | -2.14 |  | -2.32 |
| *S100a14* |  | -1.92 |  | -2.32 |
| *Scel* |  | -2.77 |  | -3.02 |
| *Scel* |  | -2.68 |  | -3.02 |
| *Scel* |  | -2.77 |  | -2.96 |
| *Scel* |  | -2.68 |  | -2.96 |
| *Serpina9* |  | -2.70 |  | -2.68 |
| *Serpinb12* |  | -5.10 |  | -5.09 |
| *Serpinb2* |  | -3.07 |  | -3.14 |
| *Serpinb3A* |  | -3.99 |  | -4.42 |
| *Serpinb5* |  | -3.42 |  | -3.19 |
| *Sfn* |  | -1.24 |  | -1.69 |
| *Sim2* |  | -2.00 |  | -2.33 |
| *Slc4a9* |  | -2.83 |  | -2.85 |
| *Slpi* |  | -2.09 |  | -2.24 |
| *Slpil2* |  | -1.74 |  | -1.64 |
| *Sox2* |  | -1.90 |  | -2.35 |
| *Spink5* |  | -2.54 |  | -2.16 |
| *Sprr3* |  | -3.75 |  | -4.23 |
| *Stfa3* |  | -5.32 |  | -4.89 |
| *Tgm1* |  | -2.53 |  | -3.12 |
| *Tmprss11a* |  | -2.97 |  | -3.12 |
| *Trim29* |  | -2.28 |  | -2.71 |
| *Wfdc12* |  | -3.13 |  | -3.30 |
| *Wfdc5* |  | -2.78 |  | -3.22 |
| *Vpreb2* |  | -1.27 |  | -1.73 |
| *Vsig8* |  | -2.58 |  | -2.31 |
| **Thyroid proteins** |  |  |  |  |
| ACTR2 |  | 1.57 |  | 1.62 |
| ACY1A |  | 1.76 |  | 1.54 |
| ADA |  | -5.31 |  | -5.69 |
| ALAD |  | -1.83 |  | -1.77 |
| AMBP |  | 1.97 |  | 1.56 |
| ANXA8 |  | -2.07 |  | -1.60 |
| APOA4 |  | -1.59 |  | -1.54 |
| APOC1 |  | -1.72 |  | -2.01 |
| ATIC |  | 1.53 |  | 1.53 |
| ATP5I |  | -1.93 |  | -1.86 |
| BCAM |  | 1.87 |  | 1.84 |
| BLOC1S5 |  | -1.85 |  | -1.82 |
| C1QBP |  | -1.64 |  | -1.64 |
| CAPZA1 |  | 1.65 |  | 1.60 |
| COX5A |  | -1.94 |  | -1.55 |
| COX6C2 |  | -1.75 |  | -1.88 |
| CSRP3 |  | -3.37 |  | -2.12 |
| CTNNB1 |  | 1.79 |  | 2.02 |
| CTSC |  | 1.75 |  | 1.80 |
| CTSH |  | 1.81 |  | 1.77 |
| CTSZ |  | 1.64 |  | 1.62 |
| DNAJC3 |  | 2.37 |  | 1.76 |
| DPY30 |  | -1.56 |  | -1.69 |
| EIF3J |  | -1.85 |  | -1.59 |
| ENTPD2 |  | 1.55 |  | 1.59 |
| ESAM |  | -1.56 |  | -1.86 |
| GALNS |  | 2.26 |  | 1.77 |
| GARS |  | 1.71 |  | 1.63 |
| GCSH |  | -1.70 |  | -1.55 |
| GDI1 |  | 1.78 |  | 1.65 |
| GYG1 |  | -1.66 |  | -1.53 |
| HEXA |  | 2.15 |  | 2.10 |
| HNRNPA3 |  | 1.67 |  | 1.56 |
| HNRNPC |  | 1.59 |  | 1.59 |
| HSPE1 |  | -1.51 |  | -1.51 |
| HYOU1 |  | 2.04 |  | 1.71 |
| KRT10 |  | -2.54 |  | -1.90 |
| KRT14 |  | -5.51 |  | -4.27 |
| KRT18 |  | 1.76 |  | 1.63 |
| KRT5 |  | -2.38 |  | -1.55 |
| LGALS1 |  | -1.68 |  | -1.71 |
| MB |  | -2.87 |  | -1.95 |
| MNF1 |  | -1.79 |  | -1.52 |
| MRPS31 |  | -1.66 |  | -1.57 |
| MYL1 |  | -1.82 |  | -1.59 |
| MYL2 |  | -3.43 |  | -1.60 |
| MYLPF |  | -2.05 |  | -1.76 |
| MYO9B |  | -2.69 |  | -1.68 |
| NAGA |  | 2.15 |  | 2.05 |
| NDUFA5 |  | -1.91 |  | -1.66 |
| ORM1 |  | -1.82 |  | -1.62 |
| PCNA |  | -1.73 |  | -1.58 |
| PDIA4 |  | 1.97 |  | 1.52 |
| PDLIM7 |  | -1.51 |  | -1.64 |
| PEX19 |  | -1.86 |  | -1.63 |
| PLA2G15 |  | 1.53 |  | 1.52 |
| PPP3R1 |  | -1.84 |  | -1.65 |
| PPT1 |  | 2.12 |  | 2.06 |
| PSMA6 |  | 1.74 |  | 1.65 |
| RAB9A |  | 1.83 |  | 1.76 |
| RALB |  | 1.78 |  | 1.59 |
| RWDD1 |  | -1.64 |  | -1.63 |
| S100A6 |  | -1.65 |  | -1.63 |
| SARS |  | 1.92 |  | 1.75 |
| SEPW1 |  | -1.52 |  | -1.51 |
| SH3GL1 |  | -1.53 |  | -1.54 |
| SHOOTIN-1 |  | 1.63 |  | 1.57 |
| SKP1 |  | -1.63 |  | -1.63 |
| SLMAP |  | -2.29 |  | -1.58 |
| SMPDL3A |  | 1.74 |  | 1.55 |
| THRSP |  | -1.85 |  | -2.84 |
| TIMM9 |  | -1.66 |  | -1.75 |
| TMED7 |  | 1.89 |  | 1.82 |
| TMOD1 |  | -1.84 |  | -1.70 |
| TMOD2 |  | -1.66 |  | -1.64 |
| TOR1AIP2 |  | -1.71 |  | -1.55 |
| TOR3A |  | 1.72 |  | 1.80 |
| TPD52L2 |  | -1.79 |  | -1.58 |
| TPP1 |  | 1.70 |  | 1.56 |
| TPPP3 |  | -2.54 |  | -1.97 |
| TSC22D3 |  | -1.60 |  | -1.53 |
| TUSC5 |  | -1.71 |  | -2.51 |
| UBA5 |  | 1.55 |  | 1.62 |
| UBXN1 |  | -1.71 |  | -1.54 |
| **Plasma proteins** |  |  |  |  |
| DNAH12 |  | 1.65 |  | -1.59 |
| HP |  | 2.79 |  | -2.14 |
| OBP1F |  | -2.43 |  | -1.71 |
| PGAM2 |  | -1.54 |  | -1.75 |
| RRAGA |  | -2.60 |  | -1.60 |
| TTR |  | 2.19 |  | -1.97 |
| **50 and 500 kBq**  **Thyroid transripts** |  |  |  |  |
| *Cd300lg* |  |  | 2.19 | 1.74 |
| **Thyroid proteins** |  |  |  |  |
| BLOC1S2 |  |  | -1.59 | -1.74 |
| CALCOCO1 |  |  | -1.66 | -1.52 |
| COMP |  |  | 2.36 | 2.91 |
| Ester hydrolase C11orf54 homolog |  |  | 2.02 | 1.89 |
| GMFB |  |  | 1.98 | 2.23 |
| HBB |  |  | -1.58 | -1.52 |
| IDH2 |  |  | 1.53 | 1.52 |
| LDHB |  |  | 1.72 | 1.67 |
| NDUFS2 |  |  | 1.83 | 1.56 |
| PSMC2 |  |  | 1.78 | 1.77 |
| YBX1 |  |  | -2.32 | -1.82 |
| **Plasma proteins** |  |  |  |  |
| ATRN |  |  | -1.66 | -1.53 |
| CHGA |  |  | 1.57 | 1.71 |
| COPS4 |  |  | 3.29 | -1.83 |
| CPQ |  |  | -1.65 | -1.64 |
| CTBS |  |  | -2.06 | -1.51 |
| CYCS |  |  | -1.56 | -1.92 |
| ESYT1 |  |  | 1.60 | 2.34 |
| F7 |  |  | 1.67 | 1.68 |
| GP1BB |  |  | -1.56 | 1.52 |
| LTA4H |  |  | 1.63 | 1.53 |
| PF4 |  |  | 1.82 | 1.68 |
| PVALB |  |  | -1.60 | -1.75 |
| RHD |  |  | 1.55 | -1.60 |
| SMPX |  |  | -1.62 | -2.08 |
